# A clickable melphalan for monitoring DNA interstrand crosslink accumulation and detecting ICL repair defects in Fanconi anemia patient cells

**DOI:** 10.1093/nar/gkad559

**Published:** 2023-07-03

**Authors:** Sara Berrada, Elena Martínez-Balsalobre, Lise Larcher, Violette Azzoni, Nadia Vasquez, Mélanie Da Costa, Sébastien Abel, Gilles Audoly, Lara Lee, Camille Montersino, Rémy Castellano, Sébastien Combes, Camille Gelot, Raphaël Ceccaldi, Jean-Hugues Guervilly, Jean Soulier, Christophe Lachaud

**Affiliations:** Aix-Marseille Univ, INSERM, CNRS, Institut Paoli-Calmettes, CRCM, Marseille, France; Aix-Marseille Univ, INSERM, CNRS, Institut Paoli-Calmettes, CRCM, Marseille, France; University Paris Cité, Institut de Recherche Saint-Louis, INSERM U944, and CNRS UMR7212, Paris, France; Laboratoire de biologie médicale de référence (LBMR) “Aplastic anemia”, Service d’Hématologie biologique, Hôpital Saint-Louis, Assistance Publique Hôpitaux de Paris, Paris, France; Aix-Marseille Univ, INSERM, CNRS, Institut Paoli-Calmettes, CRCM, Marseille, France; University Paris Cité, Institut de Recherche Saint-Louis, INSERM U944, and CNRS UMR7212, Paris, France; Laboratoire de biologie médicale de référence (LBMR) “Aplastic anemia”, Service d’Hématologie biologique, Hôpital Saint-Louis, Assistance Publique Hôpitaux de Paris, Paris, France; University Paris Cité, Institut de Recherche Saint-Louis, INSERM U944, and CNRS UMR7212, Paris, France; Laboratoire de biologie médicale de référence (LBMR) “Aplastic anemia”, Service d’Hématologie biologique, Hôpital Saint-Louis, Assistance Publique Hôpitaux de Paris, Paris, France; Aix-Marseille Univ, INSERM, CNRS, Institut Paoli-Calmettes, CRCM, Marseille, France; Aix-Marseille Univ, INSERM, CNRS, Institut Paoli-Calmettes, CRCM, Marseille, France; Aix-Marseille Univ, INSERM, CNRS, Institut Paoli-Calmettes, CRCM, Marseille, France; Aix-Marseille Univ, INSERM, CNRS, Institut Paoli-Calmettes, CRCM, Marseille, France; Aix-Marseille Univ, INSERM, CNRS, Institut Paoli-Calmettes, CRCM, Marseille, France; Aix-Marseille Univ, INSERM, CNRS, Institut Paoli-Calmettes, CRCM, Marseille, France; Inserm U830, PSL Research University, Institut Curie, Paris, France; Inserm U830, PSL Research University, Institut Curie, Paris, France; Aix-Marseille Univ, INSERM, CNRS, Institut Paoli-Calmettes, CRCM, Marseille, France; University Paris Cité, Institut de Recherche Saint-Louis, INSERM U944, and CNRS UMR7212, Paris, France; Laboratoire de biologie médicale de référence (LBMR) “Aplastic anemia”, Service d’Hématologie biologique, Hôpital Saint-Louis, Assistance Publique Hôpitaux de Paris, Paris, France; Aix-Marseille Univ, INSERM, CNRS, Institut Paoli-Calmettes, CRCM, Marseille, France

## Abstract

Fanconi anemia (FA) is a genetic disorder associated with developmental defects, bone marrow failure and cancer. The FA pathway is crucial for the repair of DNA interstrand crosslinks (ICLs). In this study, we have developed and characterized a new tool to investigate ICL repair: a clickable version of the crosslinking agent melphalan which we name click-melphalan. Our results demonstrate that click-melphalan is as effective as its unmodified counterpart in generating ICLs and associated toxicity. The lesions induced by click-melphalan can be detected in cells by post-labelling with a fluorescent reporter and quantified using flow cytometry. Since click-melphalan induces both ICLs and monoadducts, we generated click-mono-melphalan, which only induces monoadducts, in order to distinguish between the two types of DNA repair. By using both molecules, we show that FANCD2 knock-out cells are deficient in removing click-melphalan-induced lesions. We also found that these cells display a delay in repairing click-mono-melphalan-induced monoadducts. Our data further revealed that the presence of unrepaired ICLs inhibits monoadduct repair. Finally, our study demonstrates that these clickable molecules can differentiate intrinsic DNA repair deficiencies in primary FA patient cells from those in primary xeroderma pigmentosum patient cells. As such, these molecules may have potential for developing diagnostic tests.

## Graphical Abstract

**Graphical Abstract:**
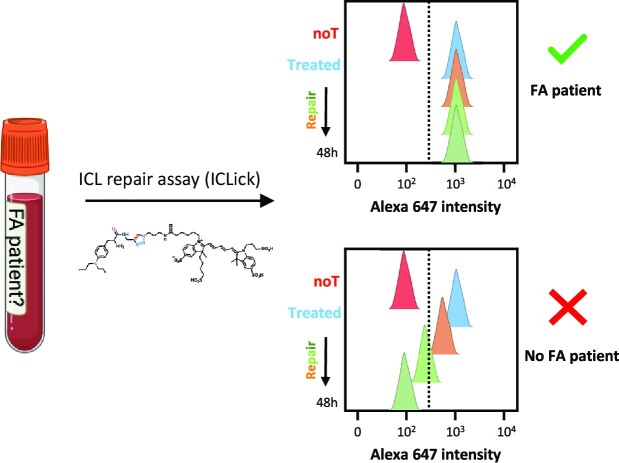

## INTRODUCTION

Interstrand crosslinks (ICLs) represent a class of highly toxic lesions that covalently link the two DNA strands, thereby blocking cellular processes such as transcription or replication. As ICLs are particularly toxic to proliferating cells, ICL-inducing agents have been used in the clinic for decades, especially for cancer therapy (1).

The repair of ICLs is extremely complex and diverse, requiring several DNA repair factors belonging to the Nucleotide Excision Repair (NER), Base Excision Repair (BER), Translesion synthesis (TLS) and homologous recombination (HR) pathways. Another central player is the Fanconi Anemia (FA) pathway, which coordinates several steps of ICL repair (as reviewed in (2). FA is a genome instability disorder associated with developmental defects, bone marrow failure and a high cancer predisposition, especially acute myeloid leukemia (AML) and head-and-neck cancers (3–6). Over the past decade, several studies suggested that endogenous reactive aldehydes could be the cause of the DNA damage (potentially including ICLs among other DNA lesions) and subsequent hematopoietic failure in FA patients ((7), reviewed in (8)). The profound cellular and chromosomal ICL hypersensitivity of FA cells has propelled the field of ICL repair.

Although replication-independent repair can occur (9–11), ICLs are assumed to be predominantly repaired in S phase. Elegant biochemical studies using Xenopus egg extracts and ICL-containing plasmids have provided important insights into the molecular mechanisms of replication-coupled ICL repair (12–18). In this model, repair of a cisplatin ICL requires the convergence of two replication forks to promote ICL unhooking, TLS and HR-dependent repair. Repair also requires the monoubiquitination of FANCD2, a critical step in the activation of the FA pathway, which promotes XPF-ERCC1-dependent unhooking of the ICL (13,15). Although very informative, this model system cannot fully illustrate the complexity of ICL repair *in vivo* within the chromatin context and the cell cycle. To study ICL repair in cells, one needs to visualize the ICL lesion and to follow its repair. In line with this, the development of digoxigenin-tagged psoralens (11,19) has provided important insight into the processing of ICLs in cells uncovering the so-called ICL traverse pathway where replication is reinitiated past the ICL leaving the lesion to be handled in a post-replicative manner (20,21). Alternatively, click chemistry was also applied to quantify psoralen-induced ICL formation and their repair (22). While psoralens generate almost exclusively ICL upon photoactivation by UVA (23), psoralen-induced ICLs turned out to be preferentially repaired through a NEIL3-dependent pathway, independent of the FA/BRCA network (17,24). Indeed, mounting evidence suggests that the nature of the ICL strongly influences the repair pathway that will be preferentially engaged (reviewed in (25)). Thus, in the context of studying FA-dependent ICL repair, the generation of other detectable ICL-inducing molecules may be more appropriate. Platinum derivatives have been developed (26–30) but mostly generate intrastrand crosslinks with ICLs only representing a small fraction of all lesions. This profile is similar for many crosslinking drugs that induce more monoadducts than ICLs (23). Hence, a strategy to differentially monitor ICLs from lesions affecting only one DNA strand is required for crosslinking agents that do not produce a majority of ICLs.

We report herein the preparation and development of the bifunctional alkylating agent melphalan and its monofunctional counterpart (mono-melphalan) modified with a small alkyne handle suitable for click chemistry (click-melphalan, click-mono-melphalan). We demonstrate that our probes have similar activities compared to unmodified melphalan molecules and can be used to differentially monitor lesion accumulation and repair in cells during the different cell cycle phases. Unexpectedly, we reveal that unrepaired ICLs negatively affect the repair of monoadducts. Interestingly, we show the efficacy of combining click-melphalan and click-mono-melphalan for the detection of ICL repair defects in FA patient primary cells. Finally, our method can also detect the NER defect in xeroderma pigmentosum (XP) patients.

Altogether, our work presents a new set of molecular probes to visualize and quantify ICL lesions and lays the foundation for their use for the diagnosis of FA patients.

## MATERIALS AND METHODS

### Chemistry-general methods

Commercially available reagents and solvents were used without further additional purification. Thin layer chromatography (TLC) was performed on precoated aluminum sheets of silica (60 F_254_ nm, Merck) and visualized using short-wave UV light. Reaction monitoring and purity of compounds were recorded by using analytical Agilent Infinity high performance liquid chromatography with DAD at 254 nM (Column Zorbax SB-C18 1.8 μM (2.1 × 50 mm), mobile phase (A: 0.1% FA H_2_O, B: 0.1% FA MeCN), flow rate 0.3 ml/min, time/%B 0/10, 4/90, 7/90, 9/10, 10/10. Column chromatography was performed on a Reveleris purification system using Reveleris Flash silica cartridges. Preparative high-performance liquid chromatography (HPLC) was carried out using an Agilent 1290 Infinity II instrument with DAD at 254nM (Column Thermo Scientific ODS Hypersil 10 μM (10 × 250 mm), mobile phase (A: 0.1% TFA H_2_O, B: 0.1% TFA MeCN), flow rate 5 ml/min, time/%B 0/10, 8/90, 12/90, 13/10, 16/10. Petroleum refers to the fraction with distillation range 40–65°C. ^1^H and ^13^C NMR spectra were recorded by using a Bruker AC 400 spectrometer. Chemical shifts, (δ) are reported in ppm and coupling values (*J*) in hertz. Abbreviations for peaks are, br: broad, s: singlet, d: doublet, t: triplet, q: quadruplet, quint: quintuplet, sex: sextuplet and m: multiplet). The spectra recorded are consistent with the proposed structures. Low-resolution mass spectra were obtained with Agilent SQ G6120B mass spectrometer in positive and negative electrospray mode. High-resolution mass spectra were performed at the Spectropole Analytical Laboratory of the University Aix-Marseille.

### (*S*)-3-(4-(bis(2-Chloroethyl)amino)phenyl)-2-((*tert*-butoxycarbonyl)amino)propanoic acid (2,31)

At 0°C, to a solution of (*S*)-2-amino-3-(4-(bis(2-chloroethyl)amino)phenyl)propanoic acid (1) (840 mg, 2.46 mmol) in methanol (60 ml) were added the triethylamine (1.37 ml, 9.84 mmol) and di-*tert*-butyl dicarbonate (1.13 ml, 4.92 mmol) successively. The resulting mixture was allowed to warm at room temperature and stirred for additional 30 min. The solvent was evaporated under reduced pressure and the residue was taken up in 0.01*N* HCl aqueous solution (50 ml) and extracted with AcOEt (3 × 30 ml). The combined organic layers were washed with brine and dried over Na_2_SO_4_. The solvent was evaporated under reduced pressure to afford the (*S*)-3-(4-(bis(2-chloroethyl)amino)phenyl)-2-((*tert*-butoxycarbonyl)amino)propanoic acid (2) as a light yellow oil which crystalized under air (1.75 g, >100%), and which was used in the following step without further purification. LC–MS C_18_H_26_Cl_2_N_2_O_4_: Rt 8256 min, ESI(+) 405.2 [M + H]^+^. ^1^H NMR (400 MHz, CDCl_3_) δ 7.06 (d, *J* = 7.9 Hz, 2H), 6.57 (d, *J* = 7.9 Hz, 2H), 5.25–5.11 (m, 1H), 4.43–4.32 (m, 1H), 3.71–3.56 (m, 8H), 3.07–2.85 (m, 2H) and 1.37 (s, 9H).

### *tert*-Butyl-(S)-(3-(4-(bis(2-chloroethyl)amino)phenyl)-1-oxo-1-(prop-2-yn-1-ylamino)propan-2-yl)carbamate (3)

To a solution of (2) (997 mg, 2.46 mmol) in *N*,*N*-dimethylformamide (6 ml) was added a solution of (benzotriazol-1-yloxy)tris(dimethylamino)phosphonium hexafluorophosphate (1.09 g, 2.46 mmol) in *N*,*N*-dimethylformamide (3 ml). After stirring at room temperature for 10 min, a solution of propargylamine (0.157 ml, 2.46 mmol) in *N*,*N*-dimethylformamide (3 ml) was injected slowly drop by drop. After addition, the solution was stirred for additional 15 min, then triethylamine (685 μl, 4.92 mmol) was added dropwise. The resulting mixture was stirred at room temperature overnight, then concentrated under reduced pressure. The residue was purified by flash chromatography, gradient DCM-MeOH (100:0 to 98:2) to afford the *tert*-butyl-(S)-(3-(4-(bis(2-chloroethyl)amino)phenyl)-1-oxo-1-(prop-2-yn-1-ylamino)propan-2-yl)carbamate (3) as a light yellow oil (970 mg, 89%). LC–MS C_21_H_29_Cl_2_N_3_O_3_: Rt 8257 min, ESI(+) 442.1 [M + H]^+^. ^1^H NMR (400 MHz, CDCl_3_) δ 7.09 (d, *J* = 8.6 Hz, 2H), 6.66 (d, *J* = 8.6 Hz, 2H), 6.06 (t, *J* = 5.2 Hz, 1H), 5.13–4.91 (m, 1H), 4.26 (q, *J* = 6.7 Hz, 1H), 3.99 (dd, *J* = 5.2, 2.5 Hz, 2H), 3.78–3.53 (m, 8H), 2.96 (d, *J* = 6.7 Hz, 2H), 2.20 (t, *J* = 2.5 Hz, 1H), 1.41 (s, 9H).

### (*S*)-2-Amino-3-(4-(bis(2-chloroethyl)amino)phenyl)-*N*-(prop-2-yn-1-yl)propanamide (4)

To a solution of (3) (970 mg, 2.20 mmol) in ether (100 ml) was added a 2*N* hydrochloric acid in ether (10 ml, 20 mmol). After addition, the stirring was maintained for 4h. The resulting mixture was extracted with ice cold water (4 × 20 ml) and combined aqueous layers were washed twice with Et_2_O (2 × 20 ml). The aqueous layer was basified with saturated Na_2_CO_3_ aqueous solution until pH 8, then extracted with DCM (3 × 20 ml). The solvent was evaporated under reduced pressure and the residue was purified by flash chromatography, gradient DCM-MeOH (100:0 to 95:5) to afford the (*S*)-2-amino-3-(4-(bis(2-chloroethyl)amino)phenyl)-*N*-(prop-2-yn-1-yl)propanamide (4) as a yellow oil which crystallized from a mixture of CHCl_3_-Et_2_O as a white powder (708 mg, 94%). LC–MS C_16_H_21_Cl_2_N_3_O: Rt 6579 min, ESI(+) 342.1 [M + H]^+^; HRMS ESI(+) calcd 342.1134 [M + H]^+^, found 342.1132; ^1^H NMR (400 MHz, MeOD) δ 7.08 (d, *J* = 8.7 Hz, 2H), 6.69 (d, *J* = 8.7 Hz, 2H), 3.97 (dd, *J* = 17.5, 2.5 Hz, 1H), 3.92 (dd, *J* = 17.5, 2.5 Hz, 1H), 3.77–3.63 (m, 8H), 3.46 (t, *J* = 6.7 Hz, 1H), 2.87 (dd, *J* = 13.5, 6.7 Hz, 1H), 2.72 (dd, *J* = 13.5, 6.7 Hz, 1H), 2.57 (t, *J* = 2.5 Hz, 1H). ^13^C NMR (101 MHz,CDCl_3_) δ 176.56, 146.55, 131.59, 127.07, 113.47, 80.44, 72.21, 57.68, 54.43, 49.00, 41.66, 29.32 (Supplementary Figure S1).

### (*S*)-2-Amino-3-(4-((2-chloroethyl)(2-hydroxyethyl)amino)phenyl)propanoic acid (5)

A solution of (1) (153 mg, 0.5 mmol) in a mixture of 1*N* hydrochloric acid aqueous solution (2 ml) and dimethylsulfoxyde (1 ml) was heated at 90°C for 50 min. The reaction was monitored by LC–MS until completion. The resulting mixture was purified without any work up by HPLC to afford the (*S*)-2-amino-3-(4-((2-chloroethyl)(2-hydroxyethyl)amino)phenyl)propanoic acid (5) as a white powder (65 mg, 45%). LC–MS C_13_H_19_ClN_2_O_3_: Rt 5.404 min, ESI(+) 287.1 [M + H]^+^; HRMS ESI(+) calcd 309.0976 [M + Na]^+^, found 309.0976; ^1^H NMR (400 MHz, MeOD) δ 7.13 (d, *J* = 8.8 Hz, 2H), 6.77 (d, *J* = 8.8 Hz, 2H), 4.15 (dd, *J* = 7.7, 5.2 Hz, 1H), 3.76–3.72 (m, 2H), 3.70–3.64 (m, 4H), 3.53 (t, *J* = 6.1 Hz, 2H), 3.20 (dd, *J* = 14.6, 5.2 Hz, 1H), 3.04 (dd, *J* = 14.6, 7.7 Hz, 1H). ^13^C NMR (101 MHz, MeOD) δ 171.41, 147.96, 131.53, 123.18, 113.86, 60.08, 55.33, 54.63, 54.54, 41.17, 36.39 (Supplementary Figure S2).

### (*S*)-2-Amino-3-(4-((2-chloroethyl)(2-hydroxyethyl)amino)phenyl)-*N*-(prop-2-yn-1-yl)propanamide (6)

A solution of (4) (337 mg, 1 mmol) in a mixture of 1*N* hydrochloric acid aqueous solution (2 ml) and dimethylsulfoxyde (1 ml) was heated at 90°C for 1h30. The reaction was monitored by LC–MS until completion. The resulting mixture was purified without any work up by HPLC to afford the (S)-2-amino-3-(4-((2-chloroethyl)(2-hydroxyethyl)amino)phenyl)-*N*-(prop-2-yn-1-yl)propanamide (6) as a white powder (130 mg, 41%). LC–MS C_16_H_22_ClN_3_O_2_: Rt 5737 min, ESI(+) 324.2 [M + H]^+^; HRMS ESI(+) calcd 324.1473 [M + H]^+^, found 324.1473; ^1^H NMR (400 MHz, MeOD) δ 7.11 (d, *J* = 8.7 Hz, 2H), 6.76 (d, *J* = 8.7 Hz, 2H), 4.01 (dd, *J* = 17.5, 2.5 Hz, 1H), 3.96 (dd, *J* = 17.5, 2.5 Hz, 1H), 3.95 (t, *J* = 7.2 Hz, 1H), 3.74 (t, *J* = 6.4 Hz, 2H), 3.70–3.63 (m, 4H), 3.54 (t, *J* = 6.0 Hz, 2H), 3.07 (dd, *J* = 14.1, 7.2 Hz, 1H), 2.95 (dd, *J* = 14.1, 7.2 Hz, 1H), 2.65 (t, *J* = 2.5 Hz, 1H). ^13^C NMR (101 MHz, MeOD) δ 169.44, 147.81, 131.58, 123.23, 113.87, 79.83, 72.79, 60.04, 55.91, 54.68, 54.57, 41.16, 37.66, 29.57 (Supplementary Figure S3).

### Cell culture

U2OS, HeLa and HeLa FANCD2 KO (32) cells were maintained at sub-confluent levels in Dulbecco's modified Eagle's medium (DMEM; Gibco) supplemented with 10% fetal bovine serum at 37°C in a humidified atmosphere containing 5% CO_2_. RPE1 and RPE1 BRCA2 KO cells (33) were maintained in DMEM-F12 (Gibco) supplemented with 10% fetal bovine serum at 37°C in a humidified atmosphere containing 5% CO_2_. Jurkat cells were maintained in RPMI 1640 Medium (Gibco) supplemented with 10% fetal bovine serum at 37°C in a humidified atmosphere containing 5% CO_2_. Primary fibroblasts culture using 4-mm-square skin biopsies were performed using local anesthesia with Emla (Astra, Rueil, France) and the Biopsy Punch (Stiefel, Rueil, France). Skin samples were thinly sliced, and the resulting fragments were adhered on plastic plates for 15 min then covered with modified Eagle medium (MEM) supplemented with antibiotic, HEPES (*N*-2-hydroxyethylpiperazine-*N*′-2-ethanesulfonic acid), MEM nonessential amino acids (Invitrogen, Cergy Pontoise, France), and 20% fetal calf serum (FCS) (GIBCO-BRL Life Technologies, Cergy Pontoise, France), at 37°C with 5% CO_2_. Individual clones were harvested after 2–3 weeks and expanded. Primary FA lymphocytes were stimulated with 100 μg/ml Phytohemagglutinin, M form (PHA-M) (Roche) during 72 h and maintained in RPMI 1640 Medium (Gibco) supplemented with 10% fetal bovine serum and at 37°C in a humidified atmosphere containing 5% CO_2_.

### Clonogenic survival analysis in HeLa cells

10^3^ HeLa cells were seeded in triplicate in 10 cm dishes and allowed to attach for 4 h before treatment for 1 hour with the indicated drugs. After 15 days, cells were washed, fixed and stained with crystal violet (Sigma). The number of colonies with >100 cells were counted. Results were normalized to plating efficiency. For each genotype, cell viability of untreated cells was defined as 100%. Data are represented as mean ± SD from three independent experiments.

### Detection of G2 arrest by flow cytometry

Low-confluence cultures of asynchronously growing HeLa cells were treated with the indicated drugs at the indicated concentration for 1h and left for 24 h in culture media. Following trypsinization, cells were collected by centrifugation, washed in PBS, and fixed in ice-cold 70% ethanol. Cells were centrifuged, washed in PBS, and treated for 15 min at 37°C with 50 μg/ml RNase A. Cells were stained at room temperature with propidium iodide (PI) solution (2 mM MgCl_2_, 10 mM PIPES buffer, 0.1 M NaCl, 0.1% Triton X-100, 0.01 mg/ml PI) and analyzed on a LSR Fortessa flow cytometer (BD Biosciences, San Jose, CA). Cell cycle profiles were created using FlowJo analysis software (Tree Star, Inc.).

### Measurement of inhibitory concentration 50 values

In antiproliferative assays, compounds were assayed for their growth inhibiting activity towards the described cancer cell lines using the CellTiter-Glo Luminesent Cell Viability Assay as described by the manufacturer (Promega Corporation). Briefly, 5 × 10^3^ cells were plated onto 96-well plates (white with clear bottom (3610, Corning Costar) with 90 μl media per well and incubated overnight. Compounds were added at different concentrations (varying from 100 to 0.032 μM) to each well and cell cultures were incubated at 37°C during 72 h. Vehicle (DMSO) was used as control and all compounds were tested in constant percentage of DMSO (0.5%). After addition of 50 μl CellTiter GLO, luminescence was measured using a Centro luminometer LB960 (Berthold). Dose–response curves were generated and 50% inhibitory concentration values (IC50) were calculated using nonlinear regression analysis (Graph Pad Prism).

### Reverse alkaline comet assay

Reverse alkaline comet assay was done as described previously (34). Briefly, HeLa cells (100 000 per well) were seeded into 6-well cluster plates and allowed to settle overnight. Cells were initially treated with DMSO vehicle or 30 μM melphalan/click-melphalan and mono-melphalan/click-mono-melphalan for 60 min and then thoroughly washed with PBS. 16 h after treatment, cell samples were treated with PBS as a control or IR (10 Gy) before harvest by trypsinization. Cells were subsequently resuspended in molten 1% Type VII low gelling temperature agarose and then allowed to set on home-made glass slides pre-coated with agarose (34). Cells were then lysed by bathing slides in ice-cold lysis buffer (100 mM Na_2_EDTA, 2.5 M NaCl, 10 mM Tris–HCl (pH 10.5), 1% Triton X-100) for 60 min and then subjected to 4 × 15 min washes with ice-cold MilliQ H_2_O. Each slide was then submerged in alkali electrophoresis buffer (300 mM NaOH, 1 mM Na_2_EDTA) for 60 min and then electrophoresed at 30 V for 30 min at 4°C. Samples were neutralized by the addition of neutralization buffer (500 mM Tris–HCl pH 7.5) for 10 min and then allowed to dry overnight at ambient temperature. Comets were stained with SYBR green I for 10 min and then washed using 3 × MilliQ H_2_O washes. Samples were visualized using a Zeiss AXIO 2 with a 20×/0.5 NA Plan Apo objective and the level of DNA damage assessed using OpenComet. At least 50 comets were scored per slide.

### Detection of FANCD2 monoubiquitination, γH2AX and p53

Low-confluence cultures of asynchronously growing HeLa cells or primary FA fibroblasts were treated with the drugs at the indicated concentration for 1h and left for 24 h in media culture for the detection of FANCD2 and γH2AX. HeLa cells were treated with 1 μM of indicated drugs for 24 h in media culture for the detection of p53. Following trypsinization, cells were washed in PBS and lysed in RIPA buffer (50 mM Tris–HCl at pH 7.5, 150 mM NaCl, 0.5% sodium deoxycholate, 0.1% SDS, 1% NP-40, 5 mM EDTA, 20 mM β-glycerophosphate, 50 mM NaF, 1X protease inhibitor (Complete EDTA-free tablet Roche), 50 U/ml Benzonase, 1 mM PMSF). Samples were separated by SDS-polyacrylamide gel electrophoresis, transferred to a membrane, and detected with anti-FANCD2 (Abcam ab108928), anti γH2AX (Cell Signaling #9718) or anti p53 (Santa Cruz Biotechnology sc-126) and ECL reagents (GE Healthcare) using Chemidoc (Biorad).

### Caspase assay

Caspase 3/7 activation was analyzed in HeLa cells treated with the indicated compounds for 16 h using the Promega Caspase Glo assay. Briefly, 5 × 10^3^ cells were plated onto 96-well plates in 100 μl of media per well 6 h before the assay. Compounds were added at 50 μM to each well, and cell cultures were incubated at 37°C for 16 h. Vehicle (DMSO) was used as a control, and all compounds were tested at a constant percentage of DMSO (0.5%). After adding 50 μl of Caspase 3/7 GLO, luminescence was measured using a Centro luminometer LB960 (Berthold).

### MMC sensitivity test

MMC-sensitivity test was performed on FA fibroblasts using a flow-cytometry based method, according to (35). Briefly, FA fibroblasts were plated in 24 multi-well plate at 10^5^ cells per well. MMC (Sigma Aldrich) was added at different concentrations and after 72 h, cells were washed, trypsinized and harvested. Propidium iodide (PI, Sigma Aldrich) was added at a final concentration of 10 mg/ml in PBS-FBS, and the fluorescence was immediately analyzed by flow cytometry after gating of the cells by standard two-parameter forward scatter (FSC; size) and side scatter (SSC; granularity), using a FACSCalibur Flow Cytometer and CellQuest analysis system (BD Biosciences). The fraction of dying cells, which allows cellular permeabilization and PI uptake was measured by a shift on FL2. By including healthy and FA control cases in the experiment, comparison of the cell sensitivity to an increasing concentration of MMC clearly discriminates the FA phenotype.

### Detection of click-melphalan and click-mono-melphalan in cells by microscopy

HeLa cells were initially seeded onto coverslips in 6-well cluster plates at 300 000 cells per well and allowed to attach 4 h. Cells were then treated with 0.5 μM of clickable molecules or DMSO vehicle for 120 min and washed with PBS. Cell samples were then pre-extracted with ice-cold CSK buffer (100 mM NaCl, 10 mM HEPES pH 7.8, 3 mM MgCl_2_, 300 mM sucrose, 0.5% Triton X-100) for 2 min on ice to remove non-chromatin bound molecules and then fixed with 4% paraformaldehyde in PBS for 10 min at room temperature. Each sample was then washed with 3% FBS in PBS and then incubated with a click reaction mixture (50 μM Alexa fluor azide 647, 1 mM copper(II) sulfate, 100 mM sodium ascorbate, 100 mM Tris–HCl pH 8.0) at room temperature for 30 min. Cells were then washed using 3 × 10 min 3% FBS in PBS rinses with mild agitation and then mounted onto glass slides in VectaShield containing 1 μg/ml DAPI. Stained samples were captured and analyzed on a Zeiss microscope equipped with a 40×/0.5 NA Plan Apo objective.

### Detection of click-melphalan and click-mono-melphalan in cells by flow cytometry

HeLa, Jurkat and FA or XP primary cells (10^6^ per dish) were seeded into 10 cm culture dishes and incubated overnight in culture medium. The following day, samples were treated with 0.5 μM click-melphalan, 0.5 μM click-mono-melphalan or DMSO vehicle for 1h. After treatment, cells were PBS-washed and released into medium. At designated time points, samples were harvested and fixed in EtOH 70%. Fixed cells were incubated in a click reaction buffer from Alexa Fluor 647 Click-iT Plus (Thermo Fisher) for 30 min at room temperature and then washed extensively using 3 × 5% FBS in PBS rinses to remove unbound reporter. Samples were then analyzed using an LSR Fortessa flow cytometer (BD Biosciences, San Jose, CA). Ten thousand events were collected for each sample and gated using forward- versus side-scatter to eliminate cell debris and doublets. The data were analyzed using FlowJo software version 7.6.5 (Tree Star, San Carlos, CA).

### Dual parameter plot assay

HeLa and HeLa FANCD2 KO cells were treated with click-melphalan or click-mono-melphalan. Detection was done with Alexa Fluor 647 Click-iT Plus (Thermo Fisher) according to manufacture recommendations and DNA was co-stained with propidium iodide. Data were collected and analyzed using LSR Fortessa flow cytometer (BD Biosciences, San Jose, CA) using 635 nm excitation and a 660/20 nm bandpass emission filter for detection of the Alexa Fluor 647 picolyl azide and 488 nm excitation and a 610/20 bandpass emission filter for detection of the propidium iodide.

### FA patient primary cells

FA patient skin fibroblast cells were cryopreserved at Saint-Louis Hospital (Paris, France). Informed consents and authorization for research and cryopreservation were given by the patients or their relatives and IRB approval from the INSERM was given under the number 12-078. Positive FA diagnosis in these patients had been performed using standard criteria including hypersensitivity to interstrand crosslinker agents in mononuclear blood cells and skin fibroblasts (35,36). Biallelic germline *FANCA* (patient EGF400) or FANCG (patient EGF390) gene mutations were further identified by sequencing fibroblast DNAs. Control, non-FA cells were obtained with informed consent from unrelated non-FA individuals.

### XP patient primary cells

The primary fibroblast cells from two known XP patients (LXP82 and LXP924), who have a homozygous deleterious *XPC* mutation (XPC c.1643_1644 delTG; p.Val548AlafsX572) (37), were cryopreserved at Saint-Louis Hospital in Paris, France. Informed consent was obtained from either the patients or the parents of minor children. These fibroblast cells did not exhibit MMC hypersensitivity or abnormal FANCD2 monoubiquitination (not shown).

## RESULTS

### A rationale for the design of click-melphalan and click-mono-melphalan

**Figure 1.**
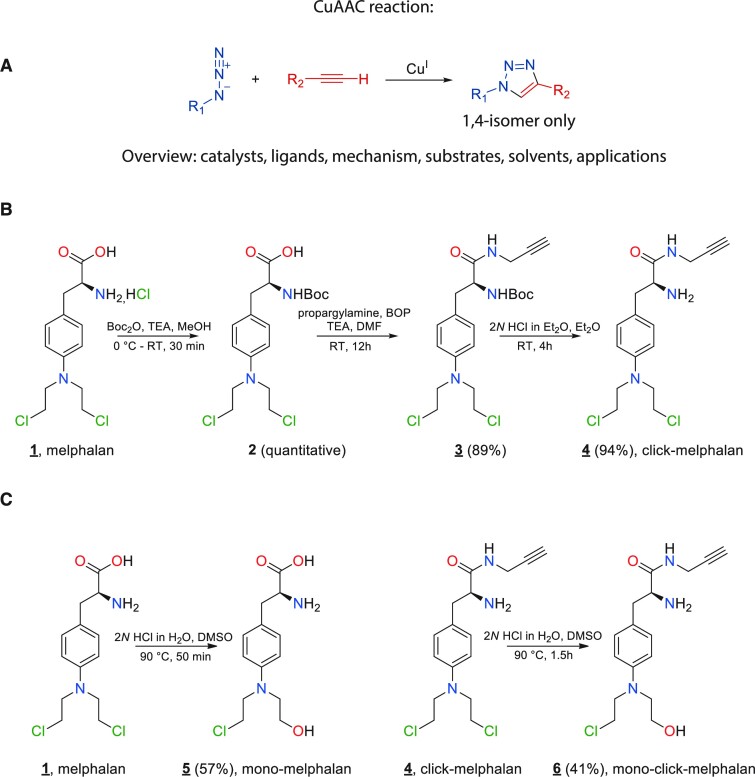
Synthesis and Labelling strategy for melphalan and mono-melphalan. (**A**) Schematic of click reaction. (**B**) Synthesis of click-melphalan 4. (**C**) Synthesis of mono-melphalan **5** and mono-click-melphalan **6**.

Click chemistry defines a group of simple high yield aqueous compatible chemical reactions that are routinely employed in biorthogonal labelling strategies to create chemical probes *in situ*. Chemical probe versions of crosslinking agents such as cisplatin and psoralen have been created using azide-alkyne cycloaddition (AAC) (38) (Figure 1A) and used as chemical probes to study DNA repair processes. Enlighten by the differentiated mode of action of melphalan, we have purposefully designed and synthesized conjugated derivatives by incorporating the clickable alkyne moiety into its chemical core. To preserve the activity of melphalan in the chemically functionalized version, we introduced the alkyne group into the carboxylic function of the melphalan molecule using a peptidic coupling reaction to generate the appropriate amide (39).

(*S*)-2-Amino-3-(4-(bis(2-chloroethyl)amino)phenyl)-*N*-(prop-2-yn-1-yl)propanamide (click melphalan, 4) was prepared in three straightforward steps from commercially available melphalan (1) (Figure 1B). The primary amine was protected using Boc_2_O to afford corresponding carbamate (2). Then, BOP-mediated peptidic coupling with propargyl amine afforded the amide (3) with a 98% yield. Removing the *N*-protecting group by standard procedure led to expected click-melphalan (4), with a purity higher than 99% and a 84% overall yield. Additionally, the syntheses of (*S*)-2-amino-3-(4-((2-chloroethyl)(2-hydroxyethyl)amino)phenyl)propanoic acid (mono-melphalan, 5) and (S)-2-amino-3-(4-((2-chloroethyl)(2-hydroxyethyl)amino)phenyl)-N-(prop-2-yn-1-yl)propanamide (click-mono-melphalan, 6) were performed from commercially available and previously prepared melphalan (1) and click-melphalan (4) respectively (Figure 1C). The compounds hydrolysis in aqueous 2*N* HCL solution at 90°C was monitored by LC–MS to avoid the formation of di-hydroxylated by-products exclusively.

### Modification for click chemistry does not change the level of melphalan-induced ICLs in cells

**Figure 2.**
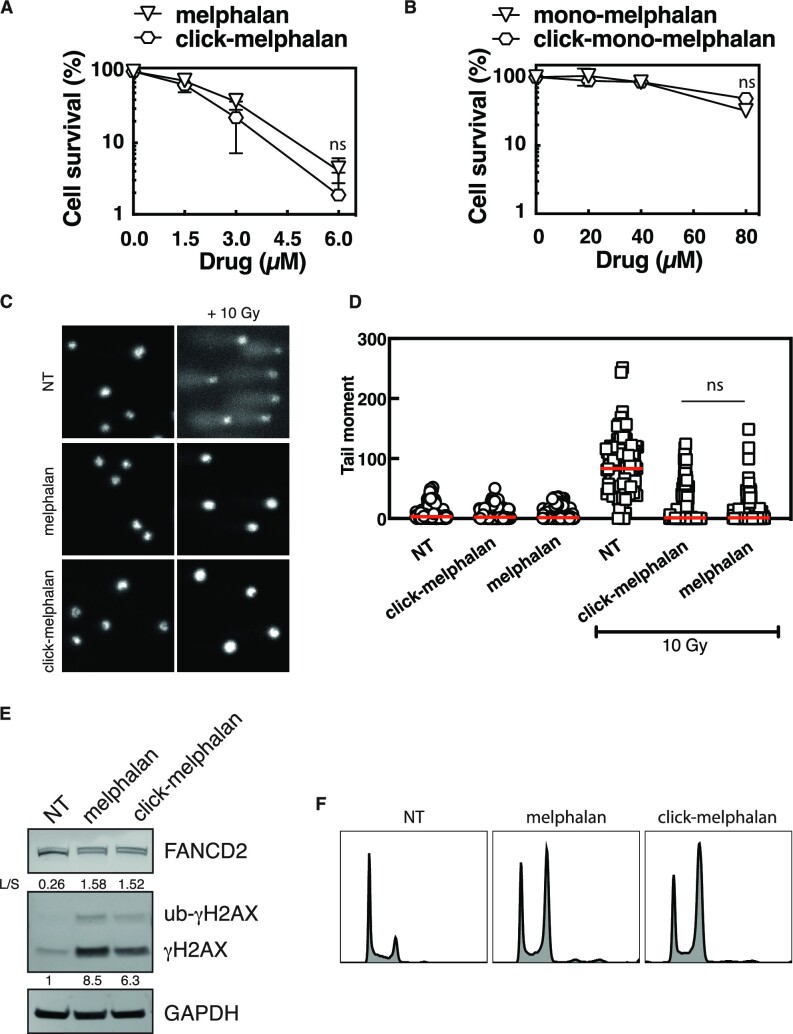
Modification for Click chemistry does not change the activities of click-melphalan or –mono-melphalan. (**A, B**) HeLa cells exposed to various concentrations of the indicated drugs were subjected to clonogenic survival assays. (**C**) Representative images of comet assays conducted on HeLa cells subjected to the indicated treatments. (**D**) Comet assay quantification indicating the decrease of the irradiation-induced comet tail moment with the indicated drugs. (**E**) Detection of FANCD2 and γH2AX in HeLa cell protein extracts after treatment with 1 μM melphalan or click-melphalan. Numbers under the blots indicate the fold induction relative to untreated samples. L/S indicates the ratio of monoubiquitinated (L, upper band) to non-monoubiquitinated (S, lower band) FANCD2. (**F**) Monitoring of cell cycle profile by propidium iodide staining in HeLa cells exposed to melphalan or click-melphalan. Data are shown as mean + s.e.m. of 3 independent experiments. ns (not significant), *P< 0.05, **P< 0.01 and ****P< 0.0001 according to two-way ANOVA followed by Šidák multiple range test (A, B) or one-way ANOVA non parametric test (D) and Mann–Whitney test (E).

To determine if the addition of an alkyne group on melphalan or mono-melphalan could affect the activity of the parent molecule, we first used clonogenic assays and compared the toxicity of click-compounds with the respective native compounds on HeLa cells and U2OS cells. Treatment of cells with both melphalan and click-melphalan (Figure 2A, Supplementary Figure S4A) or mono-melphalan and click-mono-melphalan (Figure 2B) similarly reduced the number of colonies formed. This was further confirmed by the measurement of IC50 activity (Supplementary Figure S4B). While the expected higher toxicity of melphalan compared to mono-melphalan reflects its unique ability to generate ICLs, the absence of differences in toxicity between melphalan and click-melphalan suggests that they produce a similar amount of DNA ICLs. To confirm the equivalent capacity of click-melphalan to induce ICLs, we used a modified alkaline comet assay which has previously been applied for the detection of DNA ICLs in cells (40). In the absence of IR, neither melphalan, click-melphalan, mono-melphalan nor click-mono-melphalan generated a visual comet score beyond the vehicle-treated control (Figure 2C, D, Supplementary Figure S4C). IR treatment induced a strong increase in the ‘tail moment’ of the comet, a measure of induced DNA damage. A pre-treatment with either melphalan or click-melphalan resulted in a large decrease in the IR-induced tail moment, while addition of mono-melphalan and click-mono-melphalan did not (Figure 2C, D). As the reversal of the tail moment by melphalan or click-melphalan reflects the formation of ICLs and no significant difference was observed between the two drugs, we conclude that the modification for click chemistry does not change the level of melphalan-induced ICLs in cells. Accordingly, cells deficient for ERCC1 or FANCD2 (Supplementary Figure S4D), two proteins involved in ICL repair, are sensitive to click-melphalan (Supplementary Figure S4E, F). Finally, we tested whether known cellular responses to melphalan treatment were also unaltered by the click-melphalan modification. Similar monoubiquitination of FANCD2, phosphorylation of γH2AX (Figure 2E), G2 arrest (Figure 2F) or activation of p53 and caspase activity (Supplementary Figure S4G, H) were observed in response to both the parent and modified molecules, demonstrating that melphalan and click-melphalan induce an equivalent biological response.

### DNA adducts induced by click-melphalan and click-mono-melphalan can be detected with an azide-tagged fluorescent reporter in cells via click chemistry

**Figure 3.**
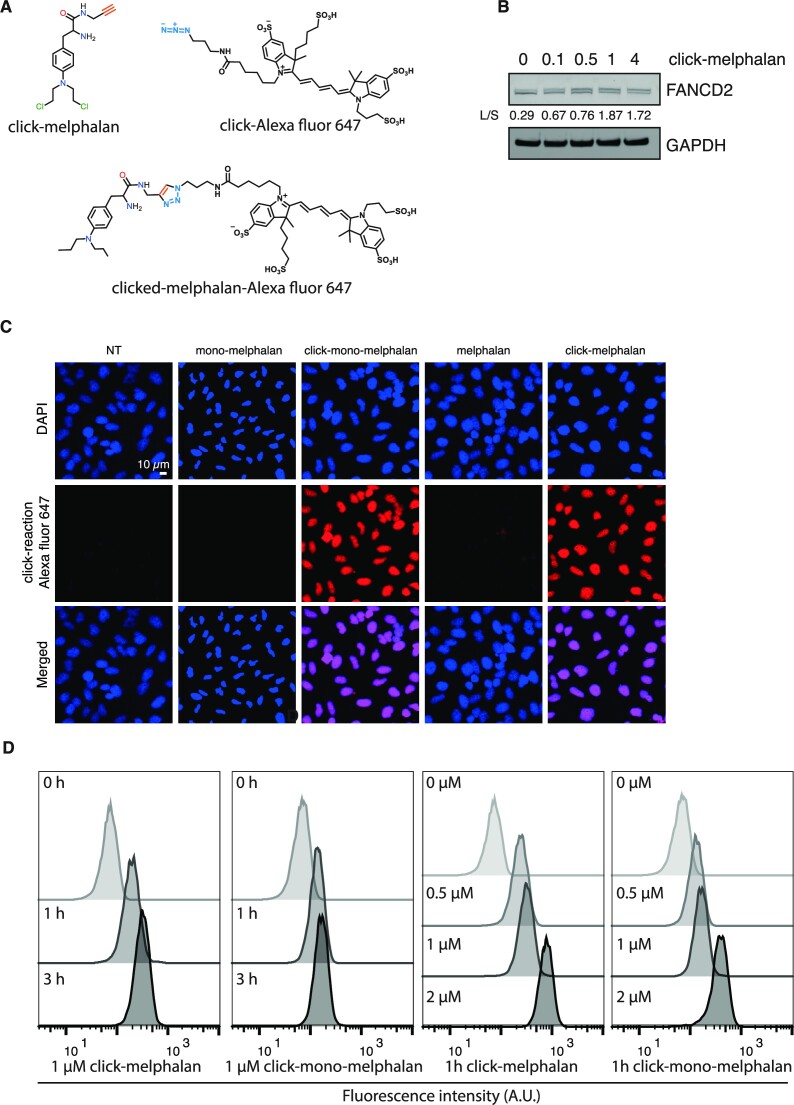
Detection of click-melphalan and click-mono-melphalan. (**A**) Schematic of click reaction between click-melphalan and Alexa fluor 647. (**B**) Detection of FANCD2 monoubiquitination with increased concentrations of click-melphalan in HeLa cells. L/S indicates the ratio of monoubiquitinated (L, upper band) to non-monoubiquitinated (S, lower band) FANCD2. (**C**) Representative images of click signals observed in HeLa cells treated with the indicated drugs. The nucleus is stained with DAPI (blue) and agents are detected by clicking an Alexa Fluor 647 azide (red). (**D**) Detection of indicated agents by Flow cytometry after click reaction with Alexa Fluor 647.

To establish whether click-melphalan and click-mono-melphalan can be used to monitor DNA lesions induced by both molecules, we conjugated them with an azide-tagged fluorescent reporter in cells (Figure 3A). We first determined that 0.5 μM was the minimum concentration of click-melphalan required to detect a robust activation of the ICL repair pathway as monitored by an increased FANCD2 monoubiquitination by western blot (Figure 3B). We then exposed HeLa cells to 0.5 μM of click-melphalan, click-mono-melphalan or unmodified melphalan. After treatment, cells were pre-extracted to remove non-chromatin bound molecules, fixed and then subjected to a click reaction mixture to enable ligation of the azide-tagged fluorescent reporter to click-melphalan and click-mono-melphalan adducts in cells. Nuclei were co-stained with DAPI prior analysis using confocal microscopy. Control samples exposed to non-modified molecules did not present significant levels of fluorescence indicating that the fluorescent reporter was not ligated to the DNA by the click reaction in the absence of clickable molecules (Figure 3C). Upon treatment with click-melphalan and click-mono-melphalan, we could detect fluorescence in cells (Figure 3C), demonstrating that the post-labelling strategy is effective for in-cell detection of clickable molecules-induced DNA lesions. The red fluorescent staining profile (middle panel, column 3–5, Figure 3C) exhibited essentially the same staining pattern as the DNA content (Top panel, Figure 3C), indicating a nuclear localization for both melphalan and mono-melphalan. We then assessed whether flow cytometry could be used to detect and quantify DNA lesions induced by our clickable molecules. After treatment with click-melphalan or click-mono-melphalan, HeLa cells were fixed and samples were subjected to a click reaction mixture with azide-tagged fluorescent reporters. Extending the time of treatment increased the level of fluorescence, indicating an increased level of DNA lesions (Figure 3D). We then applied various concentrations of clickable molecules and determined that a fluorescent signal was detectable from 0.5 μM and increasing up to 2 μM (Figure 3D). Collectively, these data validate click-melphalan and click-mono-melphalan as functional clickable probes suitable to quantify DNA lesions in cells, with the potential to monitor their repair. We named the method ICLick.

### The presence of ICL inhibits monoadduct repair

**Figure 4.**
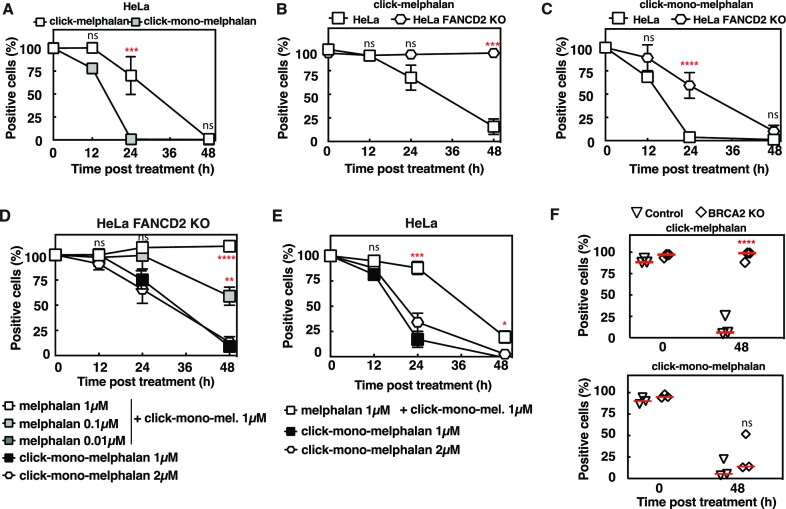
Repair of mono-melphalan and melphalan in WT and DNA repair deficient cells. (**A**) Quantification of HeLa cells positive for click-melphalan or click-mono-melphalan at the indicated time post treatment. (**B**) Same as A in HeLa and HeLa FANCD2 KO cells treated with click-melphalan. (**C**) Same as (B) with click-mono-melphalan. (**D**) Quantification of HeLa FANCD2 KO cells positive for click-mono-melphalan co-treated or not with non-clickable melphalan at the indicated time post treatment. Statistical analysis was performed in comparison with click-mono-melphalan 1 μM. (**E**) Quantification of HeLa cells positive for click-mono-melphalan co-treated or not with melphalan at the indicated time post treatment. Statistical analysis was performed in comparison with click-mono-melphalan 1 μM. (**F**) Quantification of click-melphalan and click-mono-melphalan positive cells at time 0 and 48 h after treatment in BRCA2-deficient cells. Statistical analysis was performed in comparison with control cells. Positive cells are determined as cells with a fluorescence above background fluorescence (A–F). Data are shown as mean + s.e.m. of 3 independent experiments. ns (not significant), *P< 0.05, **P< 0.01, ***P< 0.001 and ****P< 0.0001 according to two-way ANOVA followed by Šidák multiple range test (A–E) or one-way ANOVA followed by Dunn's multiple comparison test (F).

To quantitatively estimate the repair of mono- or melphalan-induced lesions, we clicked azide-tagged fluorescent reporter to unsynchronized HeLa or Jurkat cells at different times after a pulse treatment with clickable molecules. The quantification of cells positive for lesions indicates that mono-melphalan-induced lesions are repaired faster that melphalan-induced lesions in both cell lines (Figure 4A, Supplementary Figure S5A, B). Quantification of the median fluorescence intensity also confirmed a more efficient repair of lesions induced by click-mono-melphalan (Supplementary Figure S5C, D). We then investigated whether click-melphalan could be used to detect an ICL repair defect using HeLa FANCD2 KO cells. While most of WT HeLa cells have repaired melphalan-induced lesions after 48 h, all HeLa FANCD2 KO cells remained positive to melphalan (Figure 4B, Supplementary Figure S6A top panel) with no reduction of the median fluorescence intensity (Supplementary Figure S6B). Surprisingly, a delay in the repair of monoadducts induced by mono-melphalan is observed at 24 h in HeLa FANCD2 KO cells compared to HeLa cells but lesions are fully repaired in both cell lines at 48 h (Figure 4C, Supplementary Figure S6A lower panel, S6C). Considering that melphalan induces over 90% of monoadducts (23) and that monoadducts are efficiently repaired in HeLa FANCD2 KO cells at 48 h, it is thus surprising to not detect any repair of the lesions induced by melphalan in the absence of FANCD2. One possibility could be that the presence of unrepaired ICLs inhibits the repair of monoadducts. To test this hypothesis, we treated FANCD2 KO cells with both melphalan and click-mono-melphalan. In this configuration, since only mono-melphalan is clickable, only the monoadducts induced by mono-melphalan are detectable. While the addition of 0.01 μM of melphalan does not impact monoadduct repair, a decrease is observed with 0.1 μM and a complete inhibition is seen with 1 μM melphalan (Figure 4D). The repair inhibition is not the mere consequence of a higher level of monoadducts induced by the addition of melphalan, since similar kinetics of repair were observed for lesions induced by 1 or 2 μM of click-mono-melphalan (Figure 4D). Hence, we conclude that the presence of unrepaired ICLs due to FANCD2 deficiency inhibits monoadduct repair (Figure 4D).

Consistent with this hypothesis, the repair of monoadducts induced by click-mono-melphalan in WT HeLa cells is inhibited by the addition of non-clickable melphalan during the first 24 h of recovery (Figure 4E), which corresponds to the time necessary to detect a significant ICL repair (Figure 4A).

### ICLs are not repaired in BRCA2-deficient cells

We next assessed the role of homologous recombination (HR) pathway, which is important for the repair of ICL downstream of FANCD2, using a BRCA2 KO RPE1 cell line. Similarly to FANCD2-deficient cells, click-mono-melphalan-induced lesions were repaired 48 h post-treatment with no significant differences from the parental cells (Figure 4F, Supplementary Figure S6D top panel,E). However, BRCA2-deficient cells displayed a strong defect in the repair of click-melphalan-induced lesions (Figure 4F, Supplementary Figure S6D lower panel,F). These data demonstrate that our approach is an ideal set-up to examine the involvement of various DNA repair proteins in mono-adduct and ICL repair.

### ICLs are repaired in late S/G2 phase by the FA pathway

**Figure 5.**
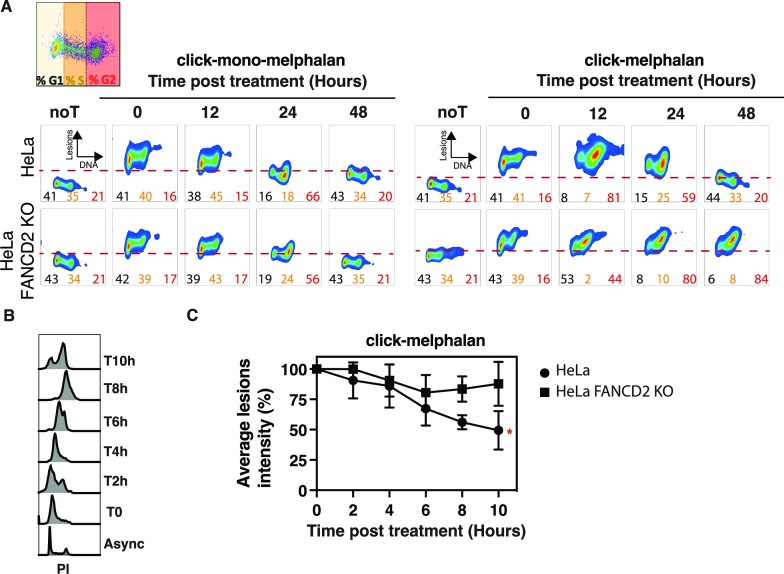
Analysis of the FA-dependent melphalan-ICL repair during the cell cycle. (**A**) Dual parameter plot of the click-mono-melphalan or melphalan subjected to the Alexa 647-click reaction with propidium iodide co-staining. Lesions repair is evaluated in HeLa and HeLa FANCD2 KO cells by flow cytometry at different time post treatment. The percentage of G1 phase cells is indicated in the left corner (black), S phase cells in the center (yellow) and G2 phase in the right corner (red). (**B**) HeLa cells were arrested in G1 phase using a double thymidine block. Cultures harvested at various time points after release were stained with propidium iodide and analyzed using flow cytometry. (**C**) Quantification of fluorescence intensity in HeLa WT or HeLa FANCD2 KO treated with click-melphalan clicked with Alexa Fluor 647 azide at the indicated time post treatment. Data are shown as mean + s.e.m. of three independent experiments. *P< 0.05 according to two-way ANOVA followed by Šidák multiple range test.

To gain insight into the timing of repair during the cell cycle, we combined click chemistry to label the lesions with propidium iodide to label DNA in HeLa and HeLa FANCD2 KO cell lines that are deficient for the FA pathway. As previously observed in Figure 4, the repair of DNA lesions induced by mono-melphalan is slightly delayed in HeLa FANCD2 KO at 24 h compared to HeLa cells. The delay is observed in all cells independently of their DNA content (Figure 5A left panel). We also detected significant differences between HeLa and HeLa FANCD2 KO cell lines in the repair of melphalan-induced lesions. At 12 h post treatment, most of the HeLa positive cells are blocked in late S-G2 and a decrease in fluorescence is observed at 24 h when cells are still blocked in late S-G2 phase with a full repair at 48 h (Figure 5A, right panel, top lane) while FANCD2 KO cells remained blocked in late S-G2 phase until 48h with unrepaired DNA lesions (Figure 5A, right panel bottom lane). These results suggest that melphalan-induced ICLs are normally repaired during late S phase in wild-type cells, in a FANCD2-dependent manner. To confirm this hypothesis, we synchronized cells at the G1/S boundary using a double thymidine block (Figure 5B, Supplementary Figure S7A). Before release into normal medium we exposed cells to click-melphalan and monitored the level of lesions in cells every 2 h using click chemistry. Under these conditions, around 50% of lesions induced by click-melphalan are repaired in HeLa cells 10h after release in S phase while no significant repair is observed in HeLa FANCD2 KO cells (Figure 5C, Supplementary Figure S7B). Our data agree with current models of ICL repair in late S phase by the FA pathway (25) and demonstrate that click-melphalan can be used to monitor ICL repair during the cell cycle.

### Click-melphalan can be used to identify ICL repair deficiency in FA patient cells

**Figure 6.**
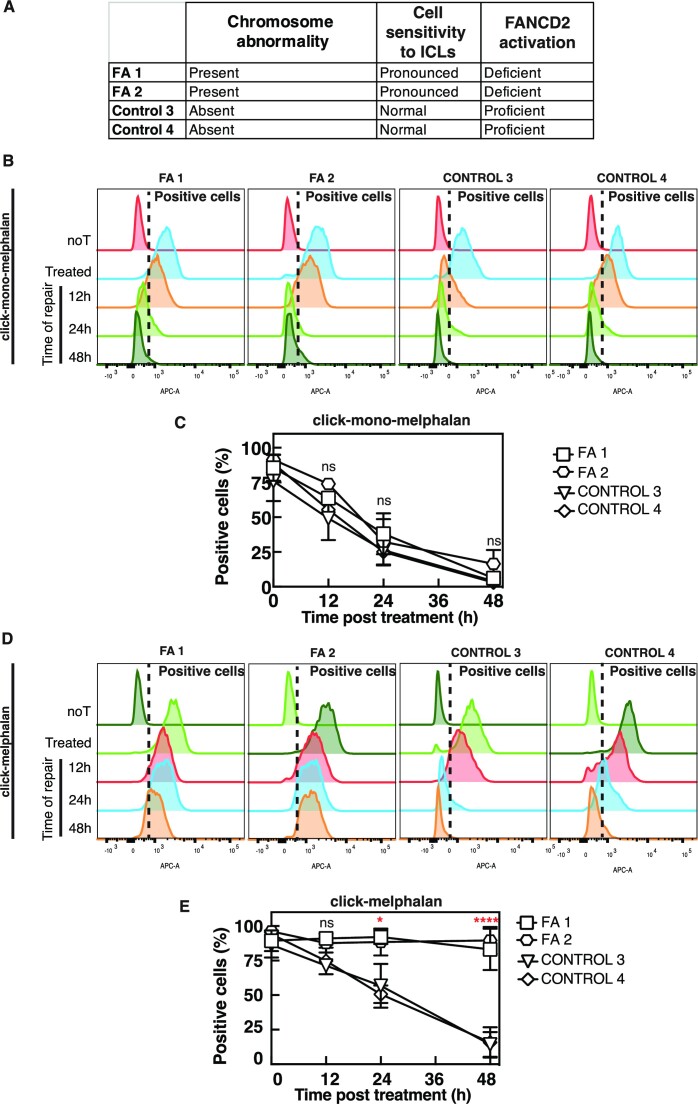
Diagnostic of FA using click-melphalan. (**A**) Table recapitulating the cellular and molecular phenotypes of cells from potential FA patients (FA 1 and FA 2) or healthy subjects (Control 3 and Control 4). (**B**) Patient cells treated with click-mono-melphalan are clicked with Alexa Fluor 647 at the indicated times post treatment and analyzed by flow cytometry. (**C**) Quantification of patient cells positive for click-mono-melphalan at the indicated time post treatment. (**D**) Patient cells treated with click-melphalan labeled with Alexa Fluor 647 at the indicated times post treatment and analyzed by flow cytometry. (**E**) Quantification of patient cells positive for click-melphalan at the indicated time post treatment. Data are shown as mean + s.e.m. of three independent experiments. ns (not significant), *P< 0.05, ****P< 0.0001 according to two-way ANOVA followed by Tukey's multiple range test.

To determine whether monitoring ICL repair with click chemistry could be used to diagnose FA we studied primary fibroblast cells from two FA patients, one FANCA (FA1) and the other FANCG (FA2) (patients EGF400#1 and EGF390#2, respectively), and compared them to two non-FA subjects (C3 and C4). As expected, FA cells but not the control cells displayed a clear hypersensitivity to ICLs and no FANCD2 monoubiquitination (summarized in Figure 6A, Supplementary Figure S8). We then monitored the repair of lesions induced by mono-melphalan or melphalan using our clickable molecules in the fibroblast cells from these four subjects. No significant differences in the reduction of signal during repair of lesions induced by mono-melphalan were observed between the FA and non-FA cells and all cells were negative for click-mono-melphalan after 48 h (Figure 6B, C). This result demonstrates that the FA cells do not display differences in their repair capacity towards DNA monoadducts. In striking contrast, lesions induced by melphalan persisted in the FA patient cells up to 48 h compared to non-FA with less than 25% cells from subject C3 and C4 still positive for melphalan compared to nearly 100% in the FA patients FA1 and FA2 (Figure 6D, E). To further evaluate the potential of our assay to diagnose FA using lymphocytes, we applied it to cryopreserved peripherical blood samples corresponding to the same patients we previously tested. Due to limited quantity of cells available, lesions were only quantified 48 h post treatment. In concordance with the results obtained in fibroblasts, PHA-stimulated lymphocytes from both FA patients were able to repair mono-melphalan- but not melphalan- induced lesions (Supplementary Figure S9).

Since the defect in repair is observed with melphalan but not with mono-melphalan, we can conclude that both fibroblasts and lymphocytes from FA patients are deficient in ICL repair. These data suggest that our molecules could support the current diagnostic regimen for FA patient by quickly identifying their defect in ICL repair.

### Click-mono-melphalan and click-melphalan can be used to identify deficiencies in xeroderma pigmentosum patient cells

Xeroderma pigmentosum (XP) is a rare inherited disorder due to a defective Nucleotide excision repair (NER) pathway. NER removes bulky DNA lesions such as those formed by UV light or some cancer chemotherapeutic agents including cisplatin and melphalan (41).

**Figure 7.**
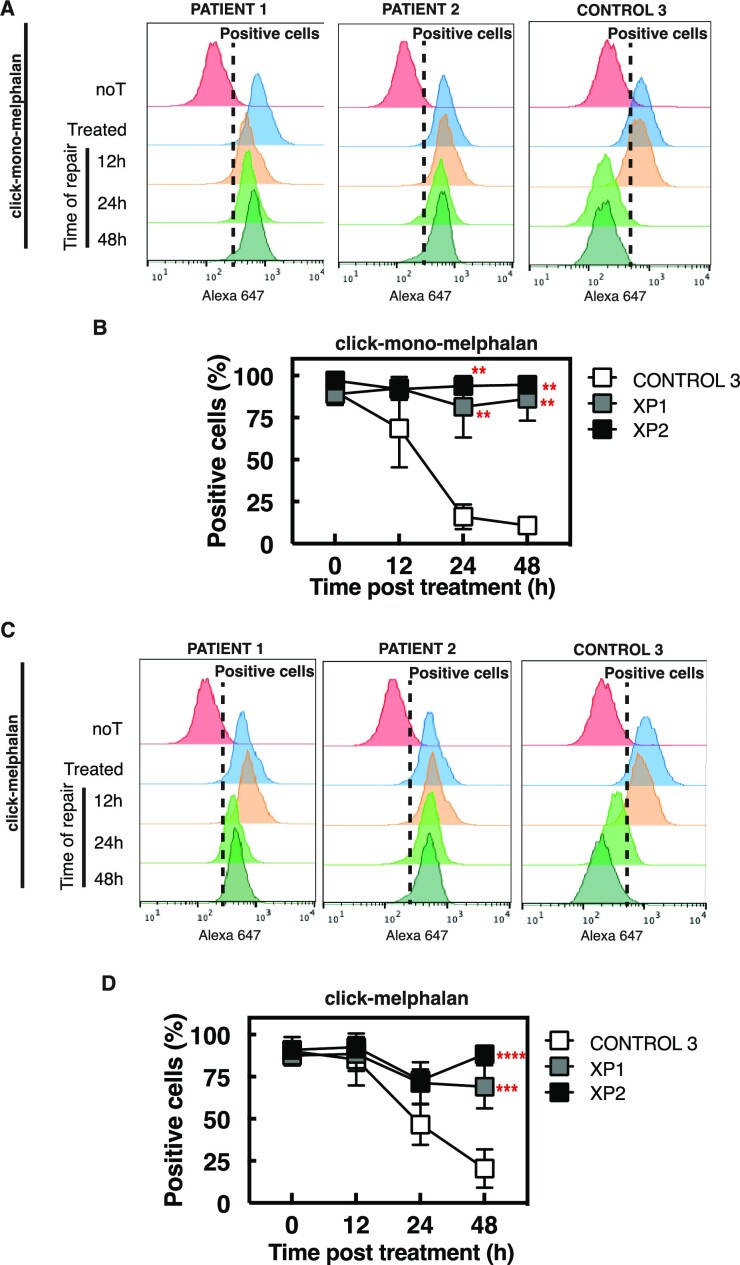
Diagnostic of Xeroderma pigmentosum (XP) patients based on click-melphalan and click-mono-melphalan detection. (**A**) Patient cells treated with click-mono-melphalan are clicked with Alexa Fluor 647 at the indicated times post treatment and analyzed by flow cytometry. (**B**) Quantification of patient cells positive for click-mono-melphalan at the indicated time post treatment. (**C**) Patient cells treated with click-melphalan labeled with Alexa Fluor 647 at the indicated times post treatment and analyzed by flow cytometry. (**D**) Quantification of patient cells positive for click-melphalan at the indicated time post treatment. Data are shown as mean + s.e.m. of three independent experiments. *P< 0.05 **P< 0.01 according to two-way ANOVA followed by Tukey's multiple range test.

Building on our work detecting ICL repair defects in FA patient cells, we wondered if our clickable molecules could also detect NER defects in fibroblast cells from two patients with XP. Lesions induced by mono-melphalan persisted in the XP patient cells up to 48 h compared to non-XP cells (Figure 7A, B), with nearly 0% of cells from subject C3 still positive for mono-melphalan compared to nearly 100% in the XP patients XP1 and XP2 (Figure 7B). Similar defects in repair were detected for lesions induced by melphalan, with 25% of cells from subject C3 still positive for melphalan compared to nearly 75% in the XP patients XP1 and XP2 (Figure 7C, D).

These data suggest that our molecules may be used to evidence syndromes other than FA due to defects in pathways involved in the repair of melphalan- or mono-melphalan-induced lesions.

## DISCUSSION

We have developed derivatives of melphalan and mono-melphalan compatible with click chemistry without impacting their intrinsic activities, thereby enabling detection of DNA lesions induced by alkylating agents in cells. We observed a fast repair of monoadducts in cells at all stages of the cell cycle, whereas ICL repair is slower and mainly occurs in late S/G2 phase in a FA-dependent manner. ICL repair coupled to DNA replication is considered to be the major ICL repair pathway (12,42–45). In the current models, ICLs are detected by the collision of the replication fork with the lesion and can be repaired either in a single fork or dual fork collision scenario (reviewed in (45)). As the probability that the two forks converge on an ICL is most likely to increase as cells progress through S phase and given that ICL repair is a slow process even *in vitro* (12), the two-fork model is fully consistent with our observation that repair of a melphalan ICL occurs in late S phase in a FA-dependent manner. More recently, Seidman and colleagues have shown using a detectable psoralen combined with DNA fiber analysis that around 20% of lesions were visualized at a stalled single fork, 15–20% were with dual fork collision and 60% of the lesions were ‘traversed’ (20). In the traversed model, replication is reprimed after the lesion leaving a X-shaped DNA molecule around the ICL, which could be repaired by the FA pathway later in phase S as we observe it. Whether fork traverse is specific to psoralen-induced ICLs or also happens at melphalan-induced ICLs could be investigated using click-melphalan.

By directly detecting the lesions induced by mono-melphalan we revealed a delay in the repair of monoadducts in HeLa FANCD2 KO cells, which can still complete repair at 48 h. This is consistent with their lack of sensitivity to mono-melphalan we observed (Supplementary Figure S4D). The FA pathway has traditionally been associated with ICL repair, but many studies have also revealed its role in modulating the response to other genotoxic stresses, with an impact on cell survival (46–48) or not (49). By being able to directly detect monoadducts, we can now monitor non-ICL lesion types and better understand their impact on replication in FA deficient cells.

Surprisingly, primary fibroblasts from patients with FA did not show a delay in the repair of mono-melphalan-induced lesions. One explanation could be a difference in monoadduct repair efficiency between HeLa cells and primary fibroblasts. For instance, repair of mono-melphalan-induced lesions is faster in HeLa cells (Figure 4A, C) compared to fibroblasts (Figure 6C), which may allow revealing the contribution of the FA pathway to monoadduct repair in HeLa FANCD2 KO cells. Another explanation could be the presence of a functional G1/S checkpoint in the primary (non-immortalized) fibroblasts preventing these from entering S-phase with unrepaired lesions and allowing pathways such as NER that act in G1 to remove the bulky monoadducts. As FANCD2 is expressed -albeit not monoubiquitinated- in the FA fibroblasts used in this work, we cannot exclude that the delay of monoadduct repair in HeLa FANCD2 KO cells could also represent a non-canonical function of unmodified FANCD2 in promoting the repair of mono-melphalan-induced lesions. This hypothesis will deserve further investigation but monoubiquitination-independent functions of FANCD2 have already been reported (50–52).

Another striking observation of our work is that the repair profile of click-melphalan-induced lesions largely differs from that of click-mono-melphalan. This is somewhat unexpected given that melphalan is estimated to generate 5–10% of ICLs among the induced DNA lesions (23). This could suggest that the proportion of ICLs induced by click-melphalan may actually be higher than anticipated. Alternatively, we postulate that the presence of unrepaired ICLs could affect the repair of monoadducts. Indeed, we observed no repair of click-melphalan-induced lesions in HeLa FANCD2 KO cells, while these cells can repair monoadducts induced by click-mono-melphalan (despite a slight delay). In agreement with this, the addition of unmodified melphalan abrogated the repair of monoadducts induced by click-mono-melphalan in FANCD2-deficient HeLa cells and delayed their repair in WT cells. The molecular basis of this observation remains to be further investigated to understand how the presence of ICLs directly or indirectly affects the repair of monoadducts, perhaps through the mobilization/sequestration of DNA repair factors at ICLs that also contribute to the repair of monoadducts or by affecting cell cycle progression.

We tested the potential of our clickable molecules to evaluate DNA repair not only in FA but also in the context of HR and NER deficiency. Our results demonstrated that a combination of click-melphalan and click-mono-melphalan is an ideal configuration for screening and detecting various DNA repair defects. This is likely to be just the ‘tip of the iceberg’, as there are other pathways involved in the ICL repair process, including mismatch repair, translesion synthesis, and base excision repair, the interplay of which still being unclear. The use of our detectable molecules in single and multiple mutants should offer the opportunity to better understand ICL repair. Another potential application would be to study the spatial distribution of ICL repair. Although it is known that ICL repair is regulated during cell cycle, it is unclear whether the (re)localization of the lesions affects their repair. While histone modifications and chromatin accessibility have established roles in regulating the repair of DNA double-strand breaks, their impact on ICL repair requires further evaluation (53). The development of the clickable cisplatin derivative APPA has shed light on the role of chromatin organization in regulating genome targeting and the spatial distribution of lesions (29). However, since cisplatin induces both intra- and interstrand crosslinks (1), which are repaired by different mechanisms, further research is required to determine the effect of chromatin organization on ICL repair.

We also hypothesize that these novel molecular probes could be used to monitor ICL repair *in vivo*. Previous detectable compounds have their limitation by either not offering the possibility to distinguish ICLs from other lesions they induce (11,22,26–29,54) or by requiring an activation by UVA (19,22). Clickable melphalan derivatives could address these limitations and provide an opportunity to evaluate how mutations in DDR genes affect ICL repair in organs or tumors. However, there is still a long way to go before being able to inject detectable ICL-inducing agents in patients, and the immediate predictive value of DNA repair in patients is limited to blood. Alkylating agents, including melphalan, are commonly used in chemotherapy but their use is limited by their high toxicity for blood cells, with around one third of patients developing hematological complications (55,56). This often results in dose reductions or treatment delays, which may compromise clinical outcome (57). One of the main causes of these side effects is the accumulation of ICL lesions that are not efficiently repaired (58), which is where assaying click-melphalan may have potential. It may potentially identify patients at risk for severe hematological complications using *ex-vivo* evaluation of the ICL repair capacity of tumor cells, allowing for the prediction of their sensitivity or resistance to treatment. Preventing discontinuation of treatment by melphalan regimen adaptation in patient with repair defect could therefore be of clinical benefit.

Another potential translational application is the use of our molecules for FA diagnosis. While FA should be suspected and searched for in all patients who develop bone marrow failure, only a fraction of them will be confirmed to have FA, the others having immunological aplastic anemia or other inherited causes (59,60). FA diagnosis is typically suspected in childhood when cytopenia occurs or based on typical malformations such as thumb abnormalities, but some individuals may not be diagnosed with FA until adulthood. Due to the large number of *FANC* genes and a large range of private mutations that may be difficult to distinguish from variants of unknown significance (VUS), it is not recommended in clinical practice to diagnose FA upfront using direct molecular tests, such as *FANC* gene sequencing, even when using gene panels or pan-genome approaches. The chromosome breakage test upon exposure to ICL-inducing agents therefore remains the current gold standard for diagnosing FA. However, this method is both labor-intensive and costly. Furthermore, there are two situations in which the chromosomal breakage test may not be conclusive. (i) at MDS or AML diagnosis when an underlying FA is suspected due to young age, physical signs or personal/family history (61); and (ii) after a first chemotherapy when an underlying FA is suspected due to unexpectedly high toxicity. In these cases, the breakage tests may not be conclusive because blast cells behave differently than lymphocytes, and confounding breaks induced by prior chemotherapy may be present, respectively. Alternative methods such as dedicated flow cytometry have been developed to analyze ICL hypersensitivity in primary fibroblast cells but these methods are still labor-intensive, time-consuming, and expensive (35). The use of click-melphalan for diagnosing or ruling out FA in blood cells could provide significant benefits to patients in terms of making informed decisions about therapeutic options and determining appropriate doses of chemotherapies, as well as selecting a suitable conditioning regimen prior to bone marrow transplantation. This finding underscores the need for further investigation in a large, prospective cohort of patients with and without FA. If proven effective, the use of click-derived diagnosis methods could offer benefits to standard methods. Beyond ICL repair and FA diagnosis, clickable melphalan derivatives may also contribute to the diagnosis of other DNA repair defects. Mono-melphalan induces DNA monoadducts that are primarily repaired by the nucleotide excision repair (NER) pathway. Defects in NER have been associated with various human syndromes including xeroderma pigmentosum (XP), Cockayne syndrome (CS) and trichothiodystrophy (TTD) (62). Our data in XP cells show the potential of click-mono-melphalan to detect a NER defect (Figure 7).

In summary, we have developed alkylating agent probes based on melphalan derivatives that can be used to monitor different defects in DNA repair through the conjugation with azide-tagged fluorescent reporters via click chemistry. This post-labelling strategy has the potential for multiple applications in clinic and for the scientific community aiming to understand ICL repair. Further studies are warranted to demonstrate the translational potential of click-melphalan.

## Supplementary Material

gkad559_Supplemental_File

## Data Availability

The cell lines, full data and molecules presented in this study are available upon request.
