# Supplementary material for: A clickable melphalan for monitoring DNA interstrand crosslink accumulation and detecting ICL repair defects in Fanconi anemia patient cells
Source: Nucleic Acids Res. 2023 Jul 3;51(15):7988–8004. doi: 10.1093/nar/gkad559 (PMC10450163; doi:10.1093/nar/gkad559)
Supplement: gkad559_Supplemental_File [file gkad559_supplemental_file.pdf]

Figure S1: (S)-2-Amino-3-(4-(bis(2-chloroethyl)amino)phenyl)-N-(prop-2-yn-1-yl)propanamide (4)

A, 1H (left) and 13C (right) NMR (400 MHz, MeOD) spectra . **B**, LC-MS chromatograms, signal DAD at 254nm (top left), MSD ES-API positive electrospray mode Total Ion Chromatogam (TIC, bottom left) and Extract Ion Chromatogram (SPC, right).

Figure S2: (S)-2-Amino-3-(4-((2-chloroethyl)(2-hydroxyethyl)amino)phenyl)propanoïc acid (5)

A, 1H (left) and 13C (right) NMR (400 MHz, MeOD) spectra . **B**, LC-MS chromatograms, signal DAD at 254nm (top left), MSD ES-API positive electrospray mode Total Ion Chromatogam (TIC, bottom left) and Extract Ion Chromatogram (SPC, right).

Figure S3: (S)-2-Amino-3-(4-((2-chloroethyl)(2-hydroxyethyl)amino)phenyl)-*N*-(prop-2-yn-1-yl)propanamide (6)

A, 1H (left) and 13C (right) NMR (400 MHz, MeOD) spectra . **B**, LC-MS chromatograms, signal DAD at 254nm (top left), MSD ES-API positive electrospray mode Total Ion Chromatogam (TIC, bottom left) and Extract Ion Chromatogram (SPC, right).

Figure S4: Toxicity of click melphalan and click-melphalan

A, U2OS cells exposed to various concentrations of the indicated drugs were subjected to clonogenic survival assays. **B,** IC50 of the indicated agents determined on HeLa cells. **C,** Comet assay quantification indicating the decrease of the irradiation-induced comet tail moment with the indicated drugs **D,** FANCD2, ERCC1 and GAPDH detection in protein extracts from HeLa WT, FANCD2 KO and ERCC1 KO cell lines. **E,** IC50 of the indicated agents determined on HeLa FANCD2 KO and ERCC1 KO cells. **F,** HeLa WT, FANCD2 KO and ERCC1 KO exposed to several concentrations of click-melphalan were subjected to clonogenic survival assays. **G,** Caspase 3/7 activation in response to high dose (50 µM) of melphalan or click-melphalan. **H,** Detection of p53 expression by western blot in Hela cells 24 h after treatment with melphalan or click-melphalan. Data are shown as mean + s.e.m. of 3 independent experiments. ns (not significant), $*p<0.05$, $**p<0.01$ and $***p<0.001$ according to two-way ANOVA followed by Šidák multiple range test (A) or by Tukey multiple range test (F) or to one-way ANOVA followed by Tukey's multiple comparison test (B, E, G) and Mann-Whitney test (C).

Figure S5: Supplementary of Figure 3

A, Jurkat or HeLa cells treated with click-mono-melphalan or click-melphalan were clicked with Alexa Fluor 647 at the indicated times post treatment and analyzed by flow cytometry. **B,** Quantification of Jurkat cells positive for lesions induced by clickable molecules at the indicated time post treatment. **C,** Quantification of fluorescence intensity in HeLa cells treated with click-melphalan or click-mono-melphalan at the indicated time post treatment. **D,** Same as C in Jurkat cells. Data are shown as mean + s.e.m. of 3 independent experiments. ns (not significant), $*p<0.05$, $**p<0.01$ and $***p<0.001$ according to two-way ANOVA followed by Šidák multiple range test.

Figure S6: Supplementary of Figure 4

A, HeLa WT and FANCD2 KO cells treated with click-mono-melphalan or melphalan were clicked with Alexa Fluor 647 at indicated times post treatment and analyzed by flow cytometry. **B,** Quantification of fluorescence intensity in WT and FANCD2 KO cell lines clicked with click-melphalan at indicated times post treatment. **C,** same as C with click-mono-melphalan treatment. **D,** Same as A in RPE1 (control) cells and BRCA2-deficient cells. **E,** Quantification of fluorescence intensity in BRCA2-KO cells treated with click-mono-melphalan. **F,** same as E with click-melphalan treatment. Data are shown as mean + s.e.m. of 3 independent experiments. *$p<0.05$, ***$p<0.001$ and ****$p<0.0001$ according to two-way ANOVA followed by Šidák multiple range test.

Figure S7: Supplementary of Figure 5

A, HeLa WT and FANCD2 KO cells were synchronized at the G1/S boundary, treated or not with click-melphalan before release into drug-free medium. Cell cycle profile was monitored by propidium iodide staining. **B,** Quantification of fluorescence intensity in HeLa WT and FANCD2 KO cell lines treated with click melphalan at indicated times post treatment.

Figure S8: Supplementary of Figure 6

A, FANCD2 ubiquitination in protein extracts from the two primary FA patient fibroblasts (FA1 and FA2) and controls**. B,** FANCD2, p53, γH2AX and GAPDH immunoblotting in protein extracts from FA1 and FA, and from healthy fibroblasts C3 and C4. **C,** Flow cytometry-based MMC sensitivity test in primary FA fibroblast from FA1 and FA2, compared to a FA and a non-FA controls. The fraction of dying cells is marked in black. The number of dying cells increases in FA cells after 1 ng/mL of MMC and in control cells after 10 ng/mL of MMC (pointed with arrows).

Figure S9: Supplementary of Figure 6

A, Patient PHA-stimulated lymphocytes treated with click-mono-melphalan were clicked with Alexa Fluor 647 post treatment or after 48 h and analyzed by flow cytometry. **B,** Quantification of patient cells positive for click-mono-melphalan.**C,** Patient cells treated with click-melphalan labeled with Alexa Fluor 647 post treatment or after 48 h and analyzed by flow cytometry. **D,** Quantification of patient cells positive for click-melphalan. Data are shown as mean + s.e.m. of 3 independent experiments. ****$p<0.0001$ according to 2way ANOVA followed by Šidák multiple range test.

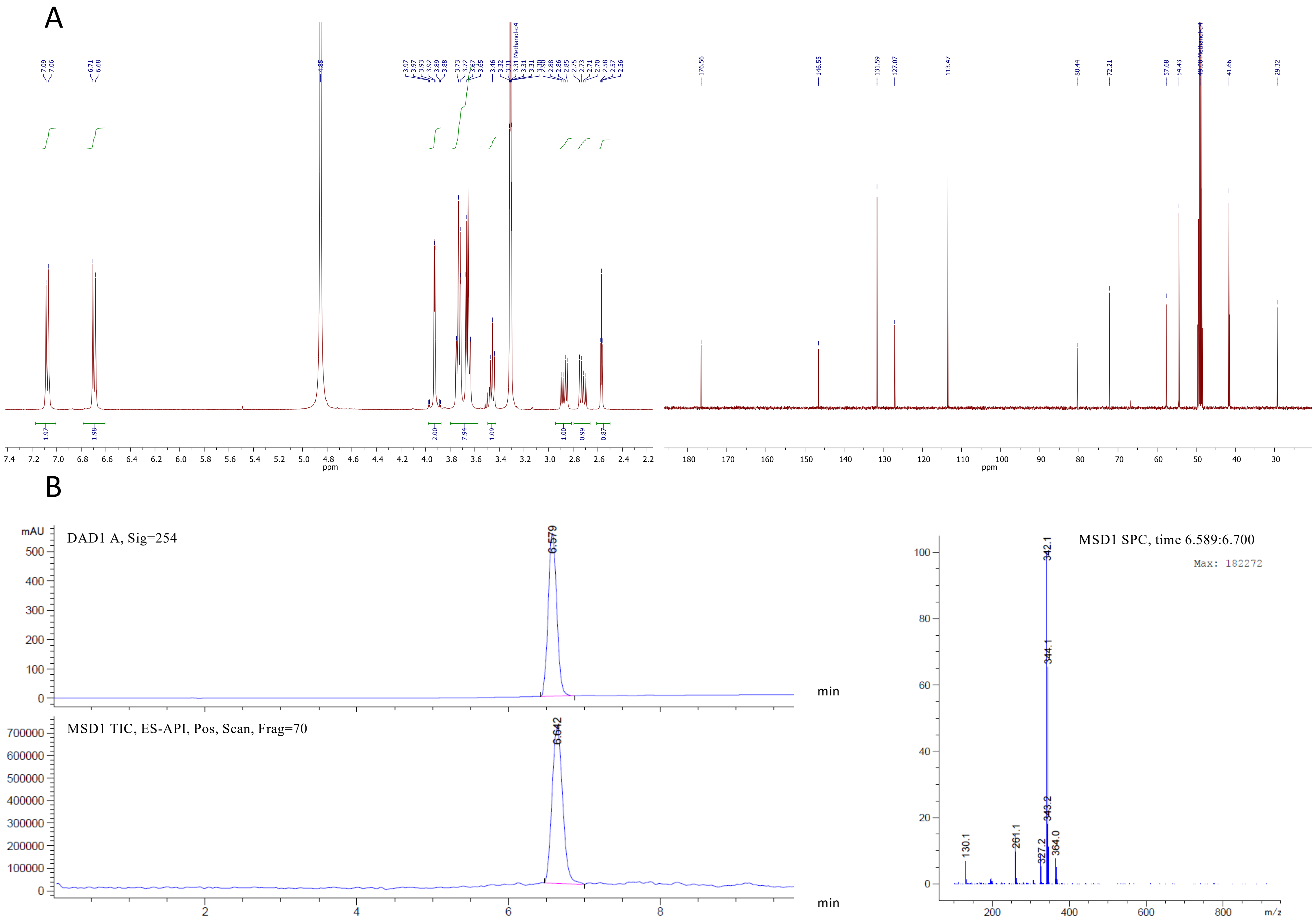

Berrada et al. Fig.S1

A

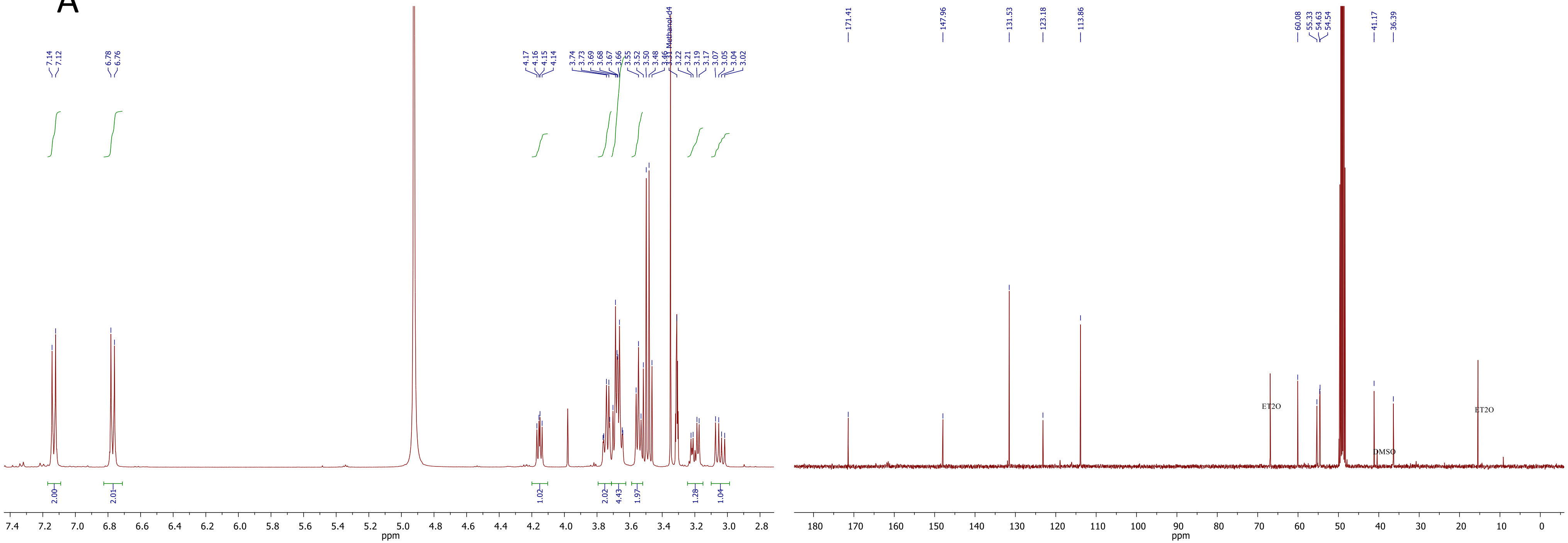

B

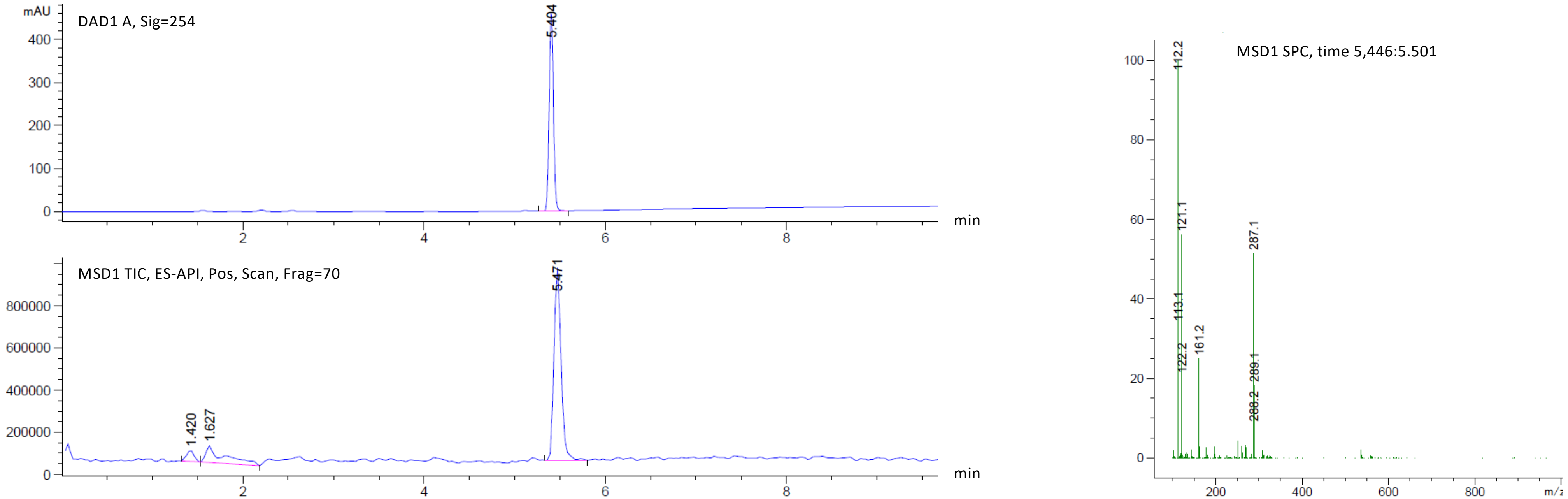

Berrada et al. Fig.S2

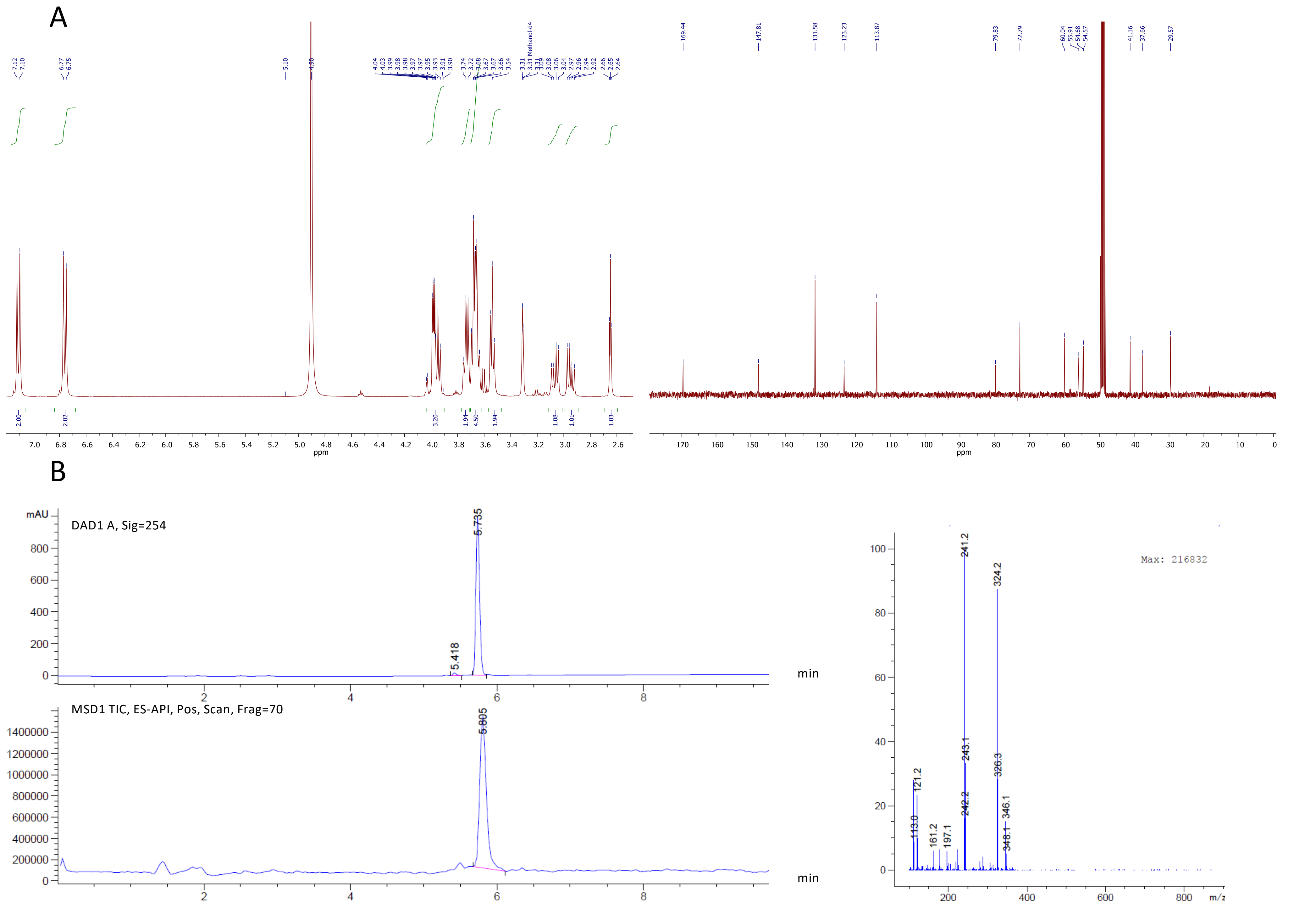

Berrada et al. Fig.S3

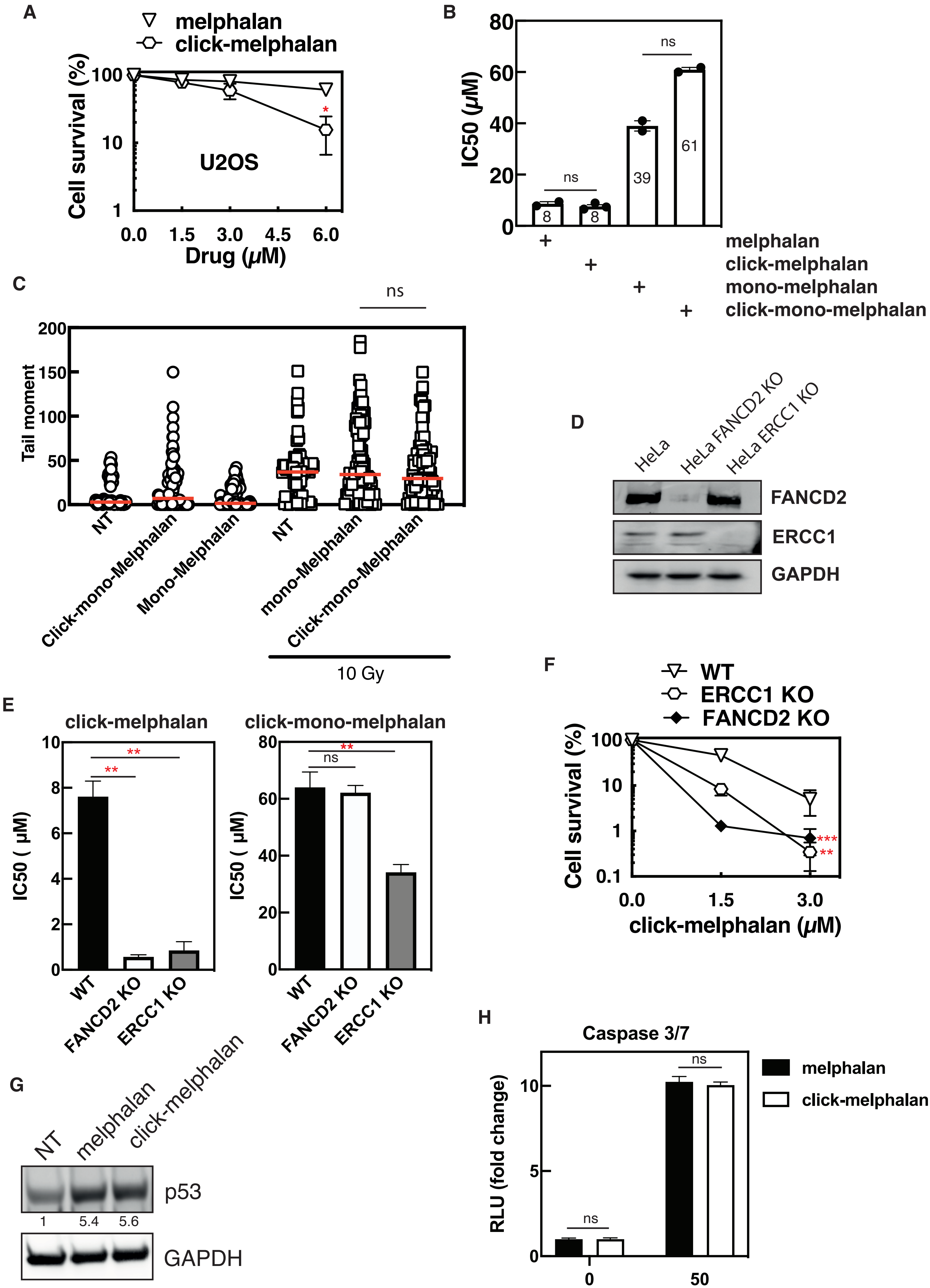

Berrada et al. Fig.S4

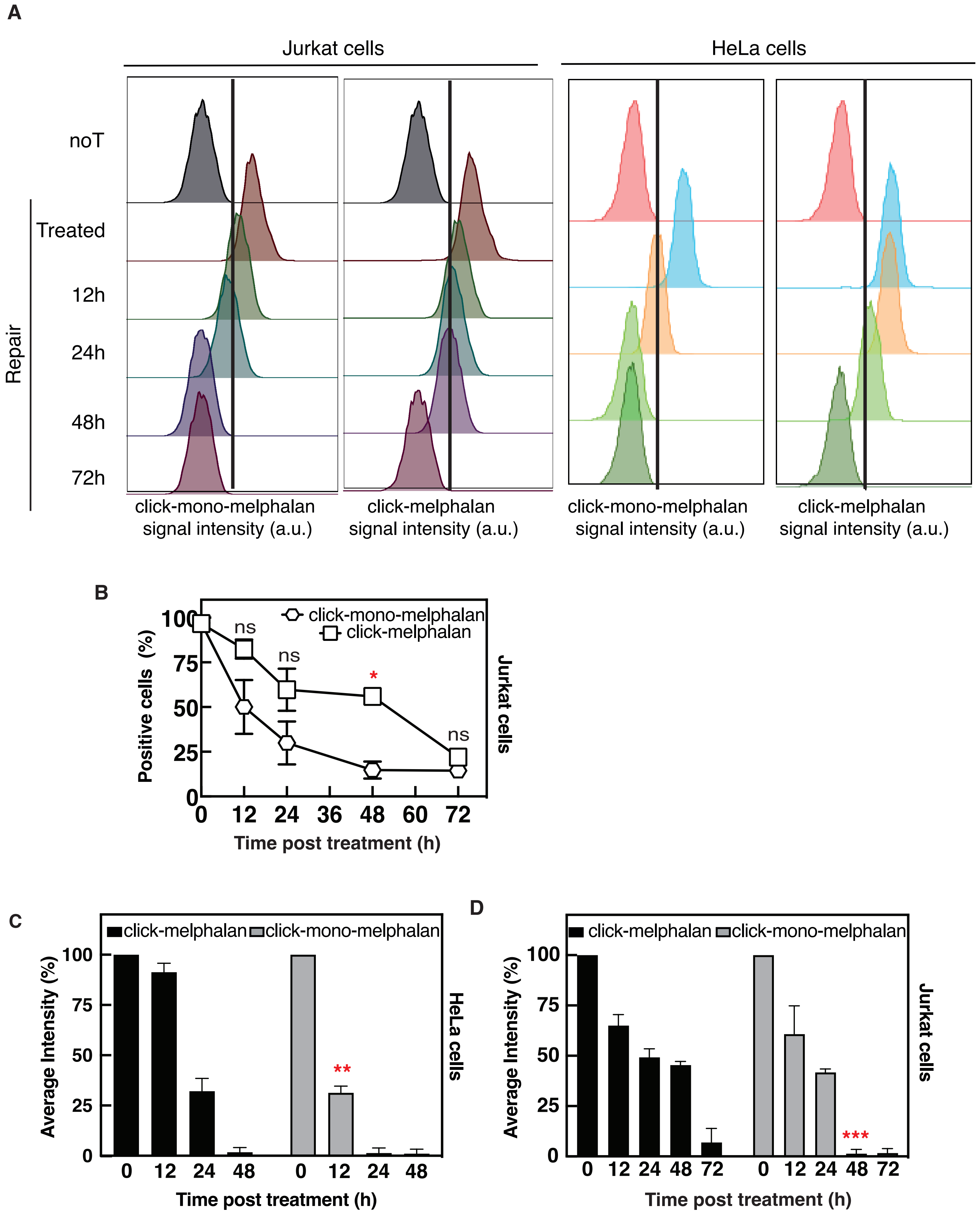

Berrada et al. Fig.S5

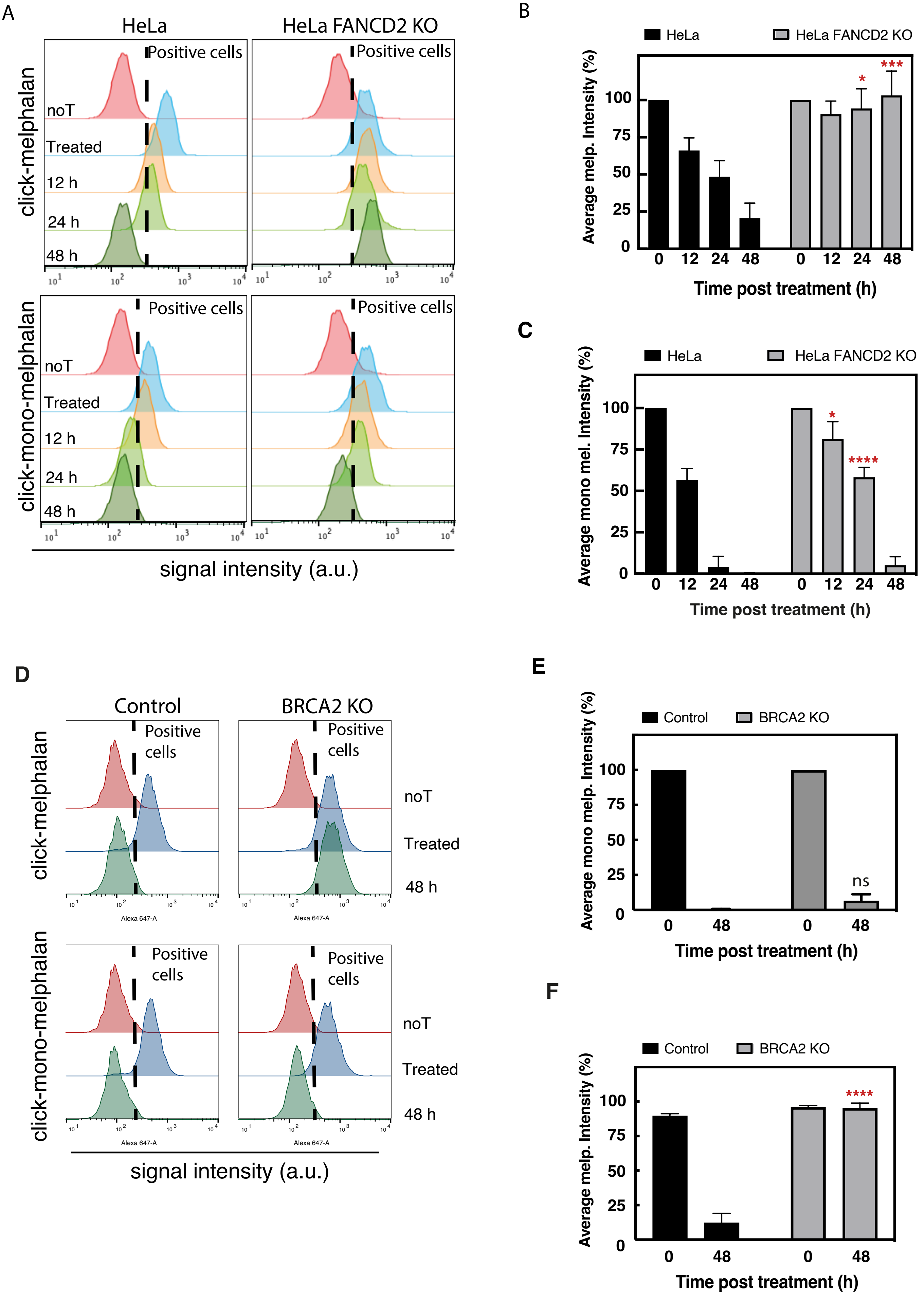

Berrada et al. Fig.S6

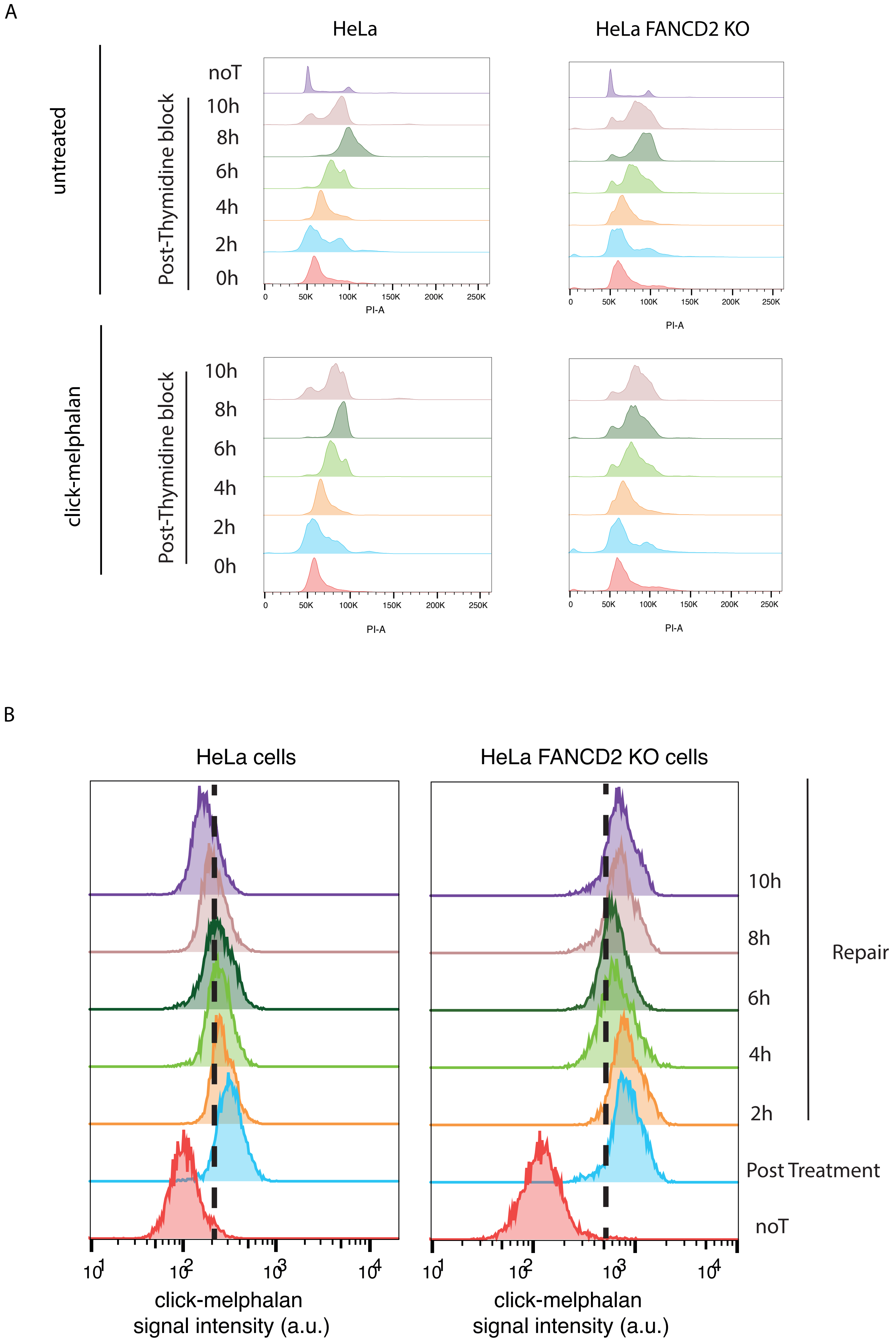

Berrada et al. Fig.S7

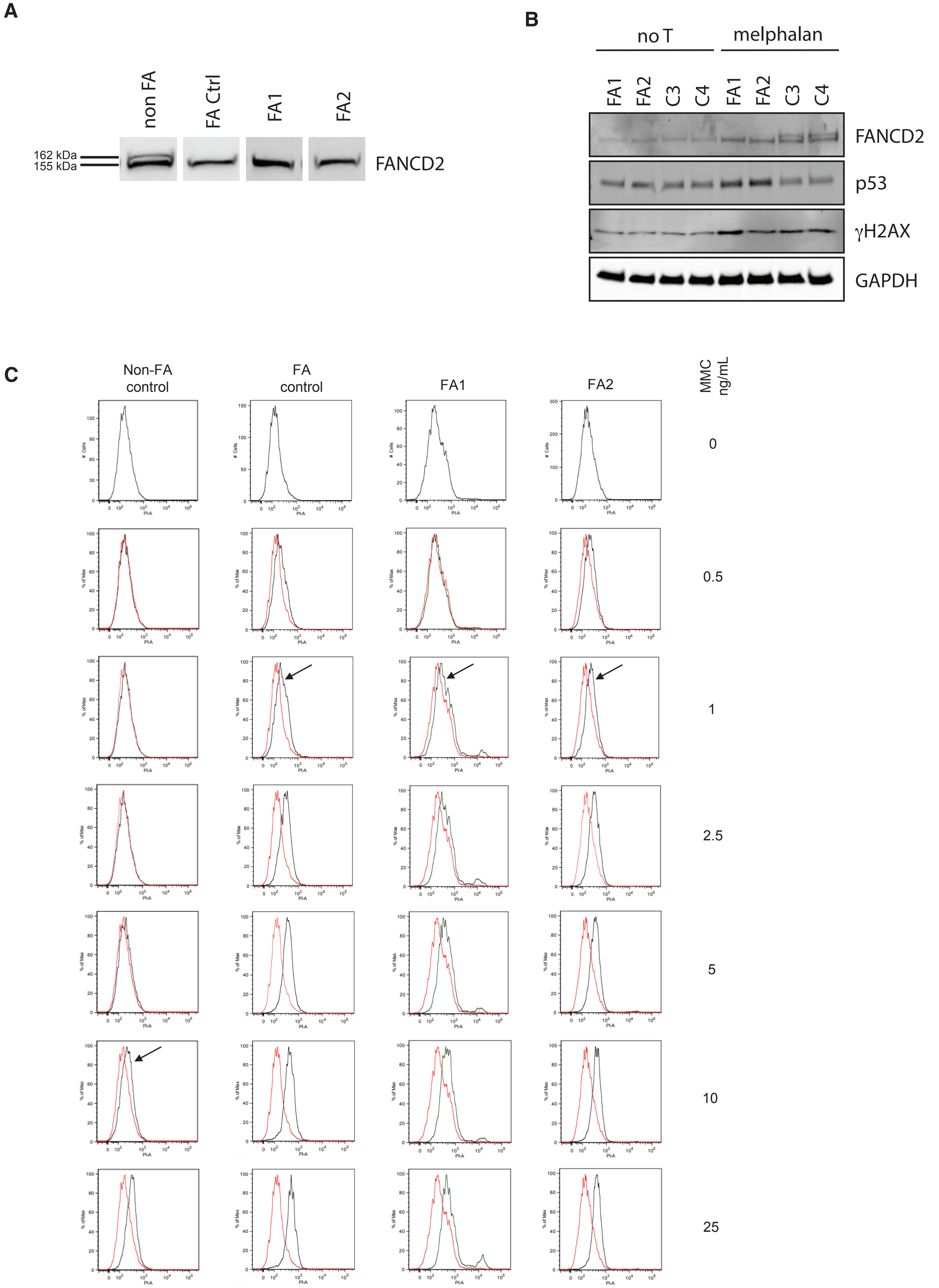

Berrada et al. Fig.S8

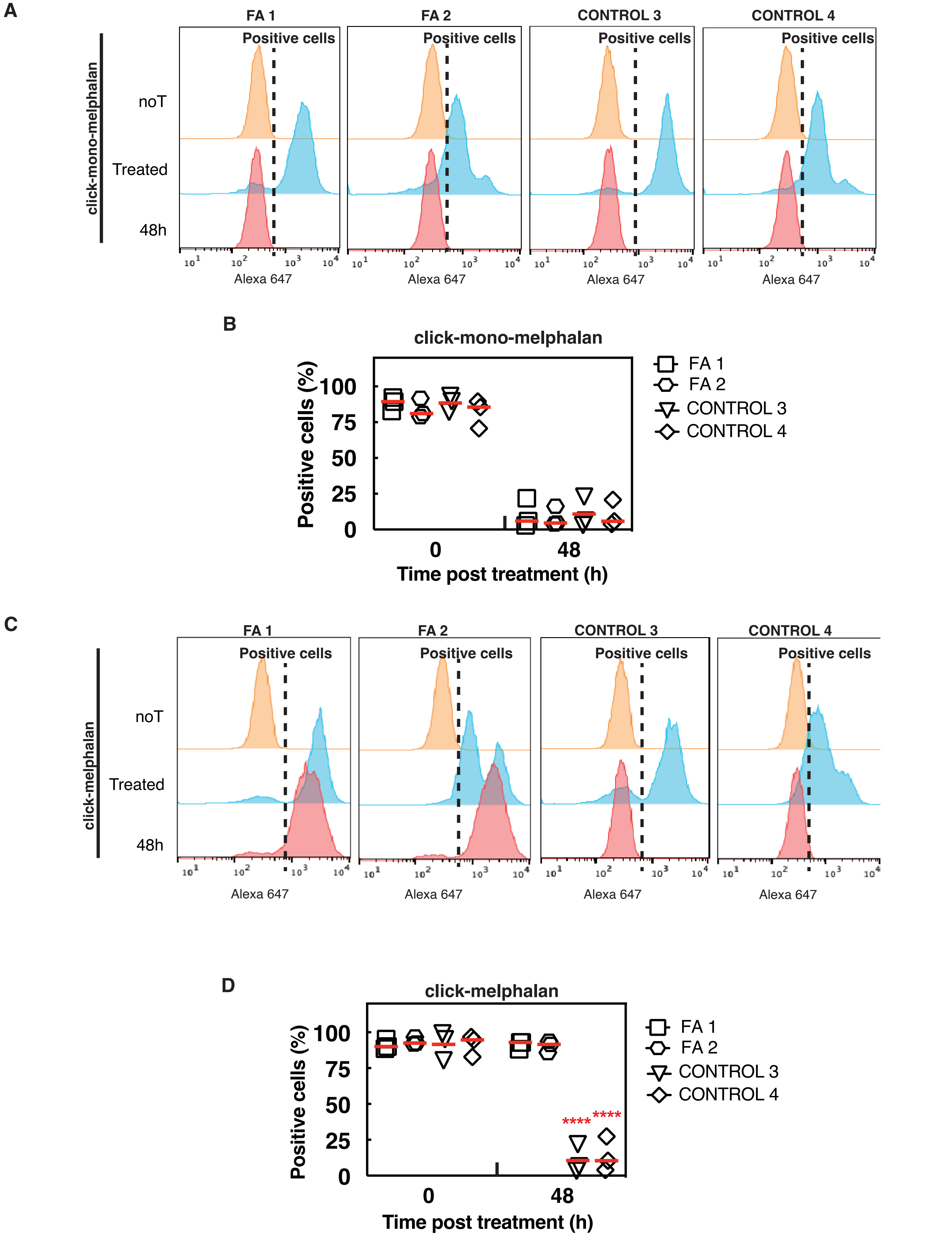

Berrada et al. Fig.S9
